# Divergent redox responses of macular and peripheral Müller Glia: Implications for retinal vulnerability

**DOI:** 10.1016/j.redox.2025.103691

**Published:** 2025-05-24

**Authors:** Ting Zhang, Kaiyu Jin, Shaoxue Zeng, Penghui Yang, Meidong Zhu, Jialing Zhang, Yingying Chen, Sora Lee, Michelle Yam, Yue Zeng, Xiaoyan Lu, Lipin Loo, G. Gregory Neely, Andrew Chang, Fanfan Zhou, Jianhai Du, Xiaohui Fan, Ling Zhu, Mark C. Gillies

**Affiliations:** a Macula Research Group, Save Sight Institute, Faculty of Medicine and Health, The University of Sydney, Sydney, NSW 2006, Australia; b Pharmaceutical Informatics Institute, College of Pharmaceutical Sciences, Zhejiang University, Hangzhou, Zhejiang, 310058, China; c New South Wales Tissue Bank, New South Wales Organ and Tissue Donation Service, Sydney, NSW, 2000, Australia; d Department of Ophthalmology, West China Hospital, Sichuan University, Chengdu, Sichuan, 610041, China; e Charles Perkins Centre, Dr. John and Anne Chong Lab for Functional Genomics, Centenary Institute and School of Life and Environmental Sciences, University of Sydney, Camperdown, New South Wales, Australia; f Save Sight Institute, Faculty of Medicine and Health, The University of Sydney, Sydney, NSW, 2000, Australia; g Molecular Drug Development Group, Sydney Pharmacy School, Faculty of Medicine and Health, The University of Sydney, Sydney, NSW 2006, Australia; h Departments of Ophthalmology and Visual Sciences and Biochemistry and Molecular Medicine, West Virginia University, Morgantown, WV, 26506, United States; i National Key Laboratory of Chinese Medicine Modernization, Innovation Centre of Yangtze River Delta, Zhejiang University, Jiaxing 314103, China

## Abstract

The macula is preferentially affected in some common retinal diseases (such as age-related macular degeneration, diabetic retinopathy and macular telangiectasia type 2), whereas most inherited retinal degenerations (e.g., retinitis pigmentosa) tend to initially affect the peripheral retina. This pattern suggests the macula may have intrinsic vulnerabilities in its oxidative stress defences, compared to the periphery. Profiling of single-cell level transcriptional changes found that the peripheral retina exhibited greater transcriptional alterations than the macula in response to stress. One pronounced change was in a subgroup of Müller glia (MG) that was dominant in the peripheral retina. Genes more abundantly expressed in peripheral MG were mainly associated with redox regulation, oxidative stress responses and cellular detoxification and were more influenced by oxidative insults, such as light-induced stress. In contrast, genes highly expressed in macular MG were primarily involved in cellular homeostasis and neuroprotection, showing less responsiveness to oxidative challenges. Notably, Metallothionein 1 (MT1), A-Kinase Anchor Protein 12 (AKAP12) and MAF BZIP Transcription Factor F (MAFF) were significantly more expressed in peripheral MG than in macular MG, indicating a region-specific redox regulatory mechanism. Knockdown of these genes in primary MG led to decreased viability under oxidative stress, suggesting their role in antioxidant defence. Our findings indicate that macular MG prioritise retinal function over redox adaptation, which may contribute to their vulnerability to degenerative diseases associated with oxidative damage. These insights underscore the importance of region-specific redox homeostasis in retinal health and disease.

## 1 Introduction

The neurosensory retina is a remarkable tissue that converts light stimuli into neurochemical signals that are transmitted to the brain for visual perception. It comprises diverse cell types, including neurons, such as photoreceptors, horizontal cells, bipolar cells, amacrine cells and retinal ganglion cells, and Müller glia which are the main glial cell of the retina. The complex interaction of neurons and glial cells maintains retinal structure and homeostasis, ensuring optimal visual function.

The retina's intense metabolic activity naturally generates reactive oxygen species (ROS), making redox (oxidation-reduction) balance essential for its health. Maintaining redox homeostasis is crucial, as disruptions in oxidative balance play a key role in the development of retinal diseases [1]. The macula, a specialized cone-rich region located at the centre of the human retina, is responsible for high-acuity central vision. This functionally important area is more prone than the peripheral retina to develop many diseases, such as age-related macular degeneration (AMD), diabetic macular edema (DME) and macular telangiectasia type 2 (MacTel) [2,3] for reasons that remain largely unknown. We hypothesise that the macula is more prone to developing disease than the peripheral retina because it is less able to mount an effective response against stress.

Müller glia, the principal glial cells of the retina that span the entire thickness of the vertebrate neural retina, play a crucial role in maintaining the structural and functional stability of the retina. They are specifically involved in regulating transcellular ion and water transport, maintaining the blood-retinal barrier and modulating neurotransmitter levels [4]. Additionally, they supply trophic factors, metabolites and antioxidants to photoreceptors and neurons, store glycogen and facilitate light transmission to photoreceptors. Müller glia can also regenerate neurons and photoreceptors in response to stress or injury in non-primate species [5]. We have previously reported that Müller glia that predominate in the macula have distinct transcriptomic profiles, as evidenced by bulk RNA sequencing results, from those that are dominant in the peripheral retina [6]. The differential response to stress of these two populations, if there is one, is yet to be clarified.

Light exposure induces age-related and oxidative stress in the retina through various mechanisms, including generating ROS, chronic inflammation, photoreceptor damage and compromised cellular repair mechanisms [[7], [8], [9]]. Light-stressed retinas in laboratory animals have been widely used in the study of retinal degeneration [9,10]. In this study, we compared the single-cell transcriptomic profiles of human retinal explants from both the macula and peripheral retina exposed to high-intensity or dim light. We hoped to gain insights into the reasons underlying the macula's particular vulnerability to developing degenerative diseases.

## 2 Results

### 2.1 Identification of human retinal cell types in the *ex vivo* cultured macula and peripheral retinas

We conducted a transcriptional analysis of 16 retinal explants from 4 *postmortem* human donor eyes **(**Supplementary Table 1**)**, comprising paired macular and peripheral retinas with and without light stress. The macular and mid-peripheral neural retinas from the left eyes were exposed to intense light (32K lux) for 4 h, while the corresponding regions from the right eyes were subjected to dim light (5 lux) as controls (Supplementary Fig. 1). We then investigated gene expression at the single-cell level (Fig. 1A).

**Fig. 1:**
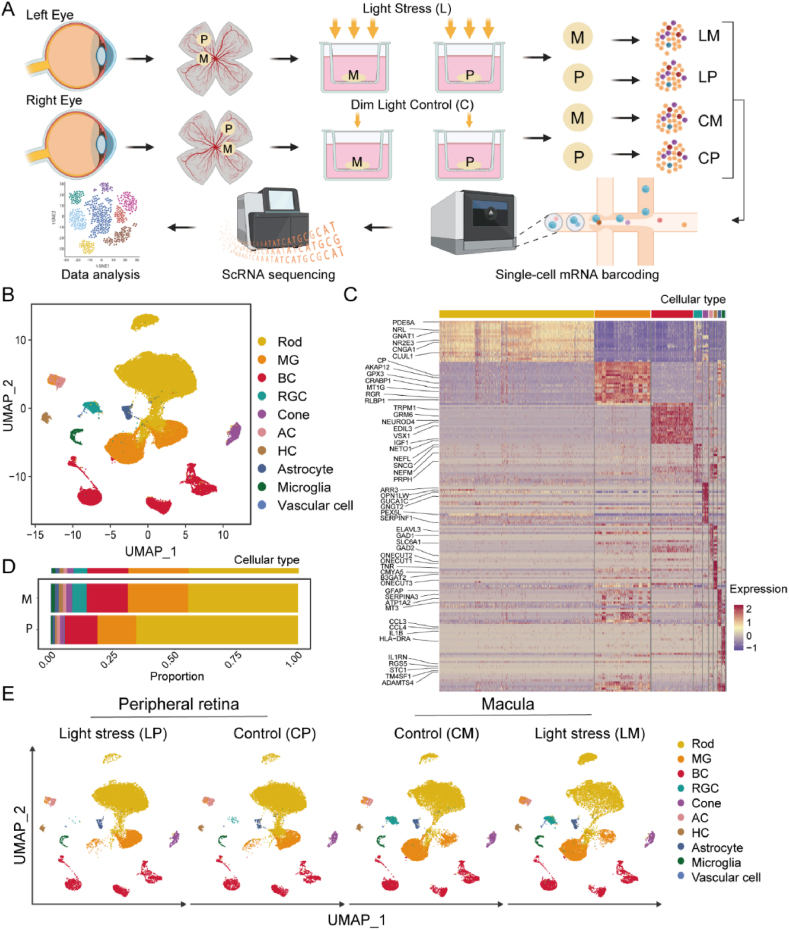
**Schematic workflow for generating single-cell retinal cells and transcriptomic profiles of retinal cells dissociated from the macula and mid-peripheral retina by single-cell RNA sequencing analysis**. **A**. The workflow illustrates the steps involved in generating single cells from human macular and peripheral retinal explants, including exposure to either bright or dim light for 4 h. The process begins with the dissection of human donor eyes to isolate the neural retina, followed by culturing the macula and mid-peripheral retinal explants on transwells. The retinal cells are dissociated from the tissue to create a single-cell suspension. n = 4 donors per treatment group. **M**: macula, **P**: peripheral retina, **L**: light stress, **C**: dim light, **LM**: macula with light stress; **LP**: peripheral retina with light stress; **CM**: macula with dim light; **CP**: peripheral retina with dim light. **B**. Retinal cell clustering using uniform manifold approximation and projection (UMAP) for all detected transcriptomes. **C**. Heatmap showing the expression of retinal cell markers in identified cell clusters. **D**. Proportions of retinal cell types in the macula and peripheral neural retinas. **E**. UMAP for the peripheral retinas (**P**) and maculas (**M**) without (**C**) or with (**L**) light stress.

We obtained 77,405 transcriptomic profiles, including 19,419 profiles from the Peripheral retinas with Light stress (**LP**), 19,887 profiles from the Peripheral retinas with dim light as the Control (**CP**), 18,290 profiles from the Macula with Light stress (**LM**) and 19,809 profiles from the Macula with dim light as the Control (**CM**). The sequencing depth (indicated by the average number of unique genes detected per cell) is illustrated in Supplementary Fig. 2A. Overall gene expression patterns were very similar across the four donors (Supplementary Fig. 2B).

We employed unsupervised clustering, uniform manifold approximation and projection (UMAP) dimensionality reduction techniques to analyse the integrated dataset. Based on the expression of established retinal cell-specific markers, we identified ten major retinal cell populations (Fig. 1B and Supplementary Fig. 2C). Each cell population was characterised by a set of unique signature genes (Fig. 1C): rods (42,687 cells), cones (1573 cells), bipolar cells (11,530 cells), retinal ganglion cells (2281 cells), amacrine cells (1071 cells), horizontal cells (965 cells), Müller glia (15,430 cells), astrocytes (943 cells), microglia (814 cells) and vascular endothelial cells (111 cells).

The cellular composition between the macula and peripheral retina was significantly different (Fig. 1D): retinal ganglion cells (5.87 % vs. 0.11 %), cones (2.32 % vs. 1.75 %) and rods (44.5 % vs. 65.5 %). We also examined the distribution and expression pattern of the cell populations across the four treatment groups (LP, CP, LM and CM) (Supplementary Fig. 2D). The proportions of cell populations in the macula and peripheral retinas did not differ between the groups exposed to bright or dim light. The degree of transcriptomic changes caused by light stress was not as great as the differences between the macula and peripheral retina (Fig. 1E).

### 2.2 Transcriptomic changes between the macular and peripheral retinal cells in response to stress

**Fig. 2:**
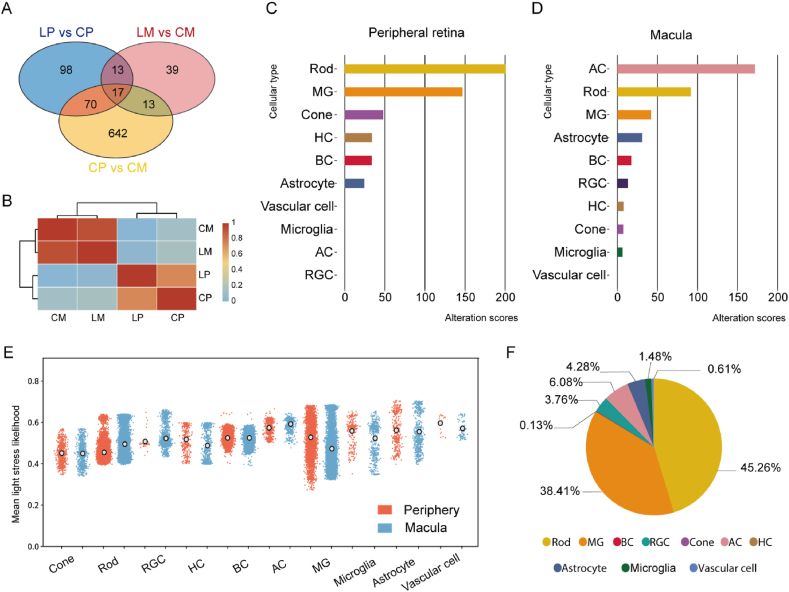
**Regional and cell type-specific alterations in response to stress**. **A**. A Venn diagram illustrates the relationships among LP vs. CP, LM vs. CM and CP vs. CM. **B**. A heatmap shows the correlations between different treatment groups. High correlation (red) between CM and LM indicates fewer transcriptional changes, while low correlation (orange) between CP and LP suggests significant transcriptomic profile changes. **LP**: Light-stressed Peripheral retina, **CP**: Control Peripheral retina, **LM**: Light-stressed Macula, **CM**: Control Macula. **C** & **D**. Alteration scores for retinal cell types in the peripheral retina (**C**) and macula (**D**) in response to light stress. Higher alteration scores indicate more significant changes. In the peripheral retina, significant changes were observed in rods and Müller glia (MG). In the macula, amacrine cells and rods exhibited the most significant changes. **E**. A scatter plot of all single cells from the macula and peripheral retina showing the distribution of light stress-associated likelihood values. Outlined circles represent average likelihood values. **F**. Proportions of each cell type among all cells from both the macula and periphery with a light stress-associated relative likelihood value greater than 0.6.

To examine the differential responses of macular and peripheral retinal cells to light stress, we assessed the gene expression changes across all retinal cells in the four treatment groups (LP, CP, LM and CM)(Supplementary Data 1). The Venn diagram (Fig. 2A) illustrates the number of genes significantly altered after light stress in the peripheral retinas (blue circle: 198 genes) and the macula (red circle: 82 genes). 30 genes were differentially expressed in both the peripheral retina and macula in response to light stress, while 742 genes were differentially expressed between the peripheral retina and macula without light stress. The correlation analysis found that the peripheral retina (LP vs. CP) displayed significantly more transcriptomic changes than macula (LM vs. CM) in response to light stress (Fig. 2B). In this heatmap, a higher correlation (red colour) represents fewer changes.

We divided the overall changes shown in the heatmap into different clusters of retinal cell types to explore cell-type-specific responses to stress in the peripheral retina (Fig. 2C) and macula (Fig. 2D). We introduced an 'alteration score' to measure changes in the quantity and expression levels of signature genes during biological processes, quantifying the extent of variation in these cell types following biological perturbation [11]. Higher alteration scores indicate more significant changes. Peripheral Müller glia and rods had significantly higher alteration scores than macular Müller glia and rods, suggesting that the peripheral retina is more responsive to light-induced stress than the macula. We observed that amacrine cells in the macula were highly sensitive to light stress, with significant and greater transcriptomic changes than amacrine cells in the peripheral retinas. We also observed that hyperglycemic stress induced more transcriptomic changes in the peripheral retina than in the macula, with higher alteration scores in peripheral Müller glia and rods compared to their macular counterparts (Supplementary Fig. 3). These findings suggest a generalised divergence in stress responses between the macula and peripheral retina.

To further explore the impact of light stress on each cell type, we calculated a light stress-associated relative likelihood (y-axis) for each cell (dots) using a manifold approximation based on all cells across both the dim light control and light stress groups (Fig. 2E). Examination of the distribution of these values across the ten previously identified cell types revealed that Müller glia had a wider range of light stress-associated relative likelihood values than other cell types (Fig. 2E). The peripheral Müller glia exhibited a higher mean value of light stress-associated likelihood compared to macular Müller glia, indicating a greater response to light stress. Fig. 2F displays the proportion of cells from each cell type in all cells from both the macula and periphery that had a high light stress-associated relative likelihood value (>0.6): these cells were mostly rods or Müller glia.

Given the known sensitivity of rod cells to light stress, understanding their response is pivotal to interpreting the broader retinal reaction. We conducted bioinformatic analyses on rod cells from both the macula and peripheral retina in response to light stress. Seven subpopulations of rod cells were identified (Supplementary Fig. 4A), with the top three subgroups constituting over 93 % of the total rod population. The distribution of these top three subgroups between the macula and peripheral retina was found to be similar (Supplementary Fig. 4B). We then identified the significantly altered genes in response to light stress within these subpopulations, categorizing them based on their macular or peripheral retinal origin. Ingenuity Pathway Analysis (IPA) revealed that despite differences in the degree of transcriptional changes, the key canonical pathways affected were largely the same, with four out of the top five pathways being identical (Supplementary Fig. 4C–D).

### 2.3 Divergent light-induced responses and biological functions of human macular and peripheral Müller glia

**Fig. 3:**
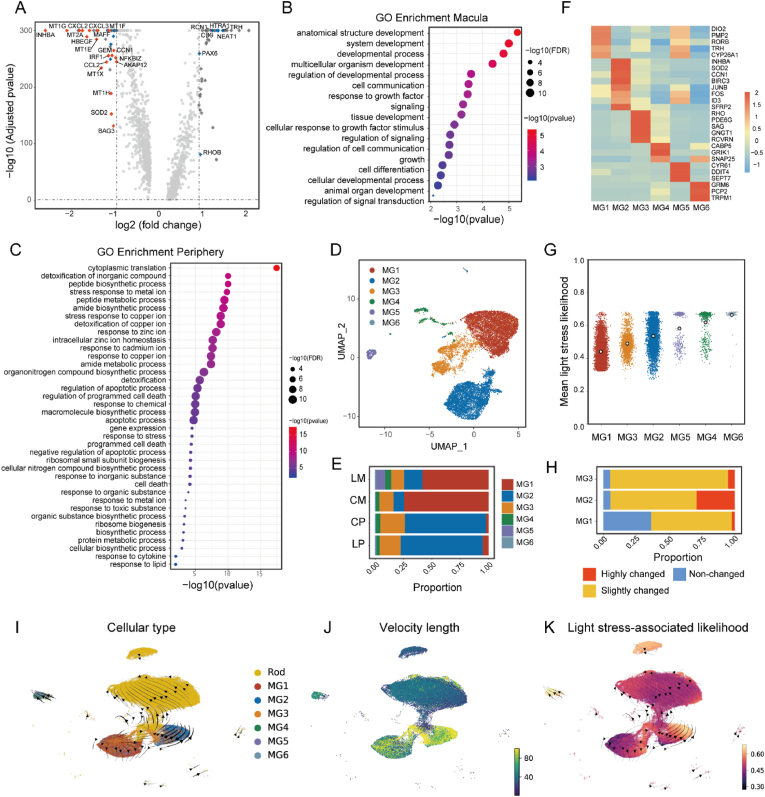
**Subclusters of human Müller glia from the macula and peripheral retinas. A**. Volcano plot of DEGs in Müller glia in the macula and peripheral retinas showing genes that were significantly differentially regulated by light stress. Red dots were upregulated while blue dots were downregulated. **B**. GO analysis of genes highly expressed in macular Müller glia (*m*-HXs) revealed significant enrichment in biological processes related to cell function and development. **C**. GO analysis of genes highly expressed in peripheral Müller glia (*p*-HXs) identified significant enrichment in various biological processes related to stress responses. **D**. UMAP of subclusters of human Müller glia identified from the macula and peripheral retinas. **E**. Proportions of Müller glia subclusters in the macula (**M**) and periphery (**P**) with (**L**) and without (**C**) light stress. **F**. Heatmap of the cluster with marker genes highly expressed in various subtypes of Müller glia. **G**. Scatter plot of six subtypes of Müller glia revealing the likelihood values of transcriptomes upon light stress. Each dot represents one Müller glia. **H**. Proportions of highly-changed (red), slightly-changed (orange) and non-changed (blue) cells in subtypes of Müller glia in response to light stress. **I**. UMAP plot of velocity and trajectory analyses revealed transition dynamics from Müller glia to rods. Colours indicate different cell types. **J**. UMAP plot of velocity length displaying the speed of differentiation. **K**. UMAP plot of single-cell velocity visualized by the previously calculated light stress-associated likelihood values.

We investigated the differentially expressed genes (DEGs) between total Müller glia from the macula and peripheral retina after exposure to light stress and hyperglycemic stress across four treatment groups (Supplementary Fig. 5A–D). The volcano plot (Fig. 3A) depicts the top 40 DEGs that were significantly more highly expressed in peripheral Müller glia compared to macular Müller glia (displayed on the left) and *vice versa* (displayed on the right). We subsequently analysed the modulation of these 80 genes under light-induced stress. Red dots in the volcano plot signify genes that were significantly upregulated (>9 %) under light stress, while blue dots indicate those that were significantly downregulated (>9 %). We found that 25 of the top 40 highly expressed genes in peripheral Müller glia (left) were significantly differentially expressed in response to light stress (19 genes upregulated and 6 downregulated) in contrast to only 6 of the top 40 highly expressed genes in macular Müller glia (right) (all 6 downregulated).

We compared the gene expression profiles of macular and peripheral Müller glia to elucidate their functional differences. We identified genes with more than a 1.5-fold expression difference in either macular or peripheral Müller glia, designating them as *m*-HXs (highly expressed genes in macular Müller glia) and *p*-HXs (highly expressed genes in peripheral Müller glia), respectively (Supplementary Data 2). Subsequent Gene Ontology (GO) analysis of both *m*-HXs and *p*-HXs focused on responses to light stress (Supplementary Data 3). Interestingly, *m*-HXs did not exhibit significant GO term enrichment related to light stress responses. However, they were significantly enriched in biological processes associated with the development of anatomical structures, cell communication, signalling, differentiation, and signal transduction (Fig. 3B, Supplementary Fig. 5E). Conversely, *p*-HXs, which were differentially upregulated in peripheral Müller glia in response to light stress, showed enrichment in pathways related to stress responses, including reactions to various compounds, metal ions, stress, cell death, metabolic processes, detoxification, and regulation of cell death (Fig. 3C**,** Supplementary Fig. 5E). The specific numbers of genes involved in each enriched biological process from the GO analysis are summarized in Supplementary Data 4. To assess whether light stress induced gliosis in our model, we examined the transcript levels of glial gliosis marker GFAP, together with glial structural marker RLBP1 (CRALBP), in Müller glia (Supplementary Table 2). We found no significant upregulation of these markers under light stress compared to controls in either the macula or the peripheral retina, suggesting that the oxidative stress responses we observed occurred without triggering a pronounced gliotic reaction.

### 2.4 Different light-induced responses and biological functions of different subtypes of Müller glia

We identified 6 subtypes (MG1 to MG6) of Müller glia in the transcriptomes obtained from the LP, CP, LM and CM groups (Fig. 3D). The proportion of two major subtypes, macula-dominant Müller glia (MG1) and peripheral retina-dominant Müller glia (MG2), accounted for approximately 75 % of Müller glia in the macula and peripheral retinas (Fig. 3E). We then focused on three subtypes (MG1 to MG3) due to their high abundance; the MG4 subtype contained relatively few cells and the MG5 and MG6 subtypes were donor-specific (Supplementary Fig. 5F). We generated a heatmap to cluster the top markers that were highly expressed in various Müller glia subtypes (Fig. 3F). We also cross-referenced the highly expressed feature genes in the different Müller glia subtypes (MG1, 2 and 3) with those reported in previous studies on Müller glia. We found MG1 cells had a high expression of feature genes, such as *COL4A3*, *FGF9*, *DIO2* and *ZNF385D*, in macular Müller glia [12]. We also found that MG2 subgroup expressed genes involved in response to oxidative stress (*SOD2*, *NFKBIA*, *NFKBIZ*, *TNFAIP3*, *BIRC3* and *CD44*). Additionally, MG3 cells expressed several rod markers, *RHO*, *PDE6G* and *GNGT1,* along with traditional Müller glia markers. The proportion of MG3 cells expanded slightly within the peripheral retinal samples after exposure to light stress. This finding is consistent with the previous reports that Müller glia have stem cell characteristics that may help regenerate injured or stressed retina [5,13]. We also found that, of all the Müller glia subtypes, MG1 cells had the lowest likelihood of light stress-associated relative values (Fig. 3G), indicating that this subset was relatively unresponsive to light stress. MG2 cells had higher values of light stress-associated relative likelihood, suggesting that this population is more responsive to light stress (Fig. 3H, red, highly changed).

We performed velocity and trajectory analysis to investigate the transitional relationship between Müller glia and rods. Individual cell velocities were projected onto the UMAP embedding, which was superimposed with Seurat-defined Müller glia subtypes and rods (Fig. 3I). The Python package PAGA was employed to determine velocity-inferred directionality [14]. We observed a differential trajectory between Müller glia and rods. MG3 exhibited the highest root-like potential and a fast pace of differentiation (Fig. 3I). This pace was maintained during the production of MG2 cells with high light stress-associated relative likelihood values, which substantially slowed down as the cells became more rod-like (Fig. 3J and K, Supplementary Fig. 5G). These findings indicate a transition trajectory that starts with control-like MG3 cells, followed by light-affected MG3 cells that ultimately result in three different endpoints (MG1, MG2 cells and rods). Remarkably, the light stress associated likelihood increases in correlation with the differential trajectory observed within these three endpoints. However, MG2 cells displayed the most rapid transition pace.

### 2.5 Target genes involved in the response to light stress in the periphery-dominant Müller glia

**Fig. 4:**
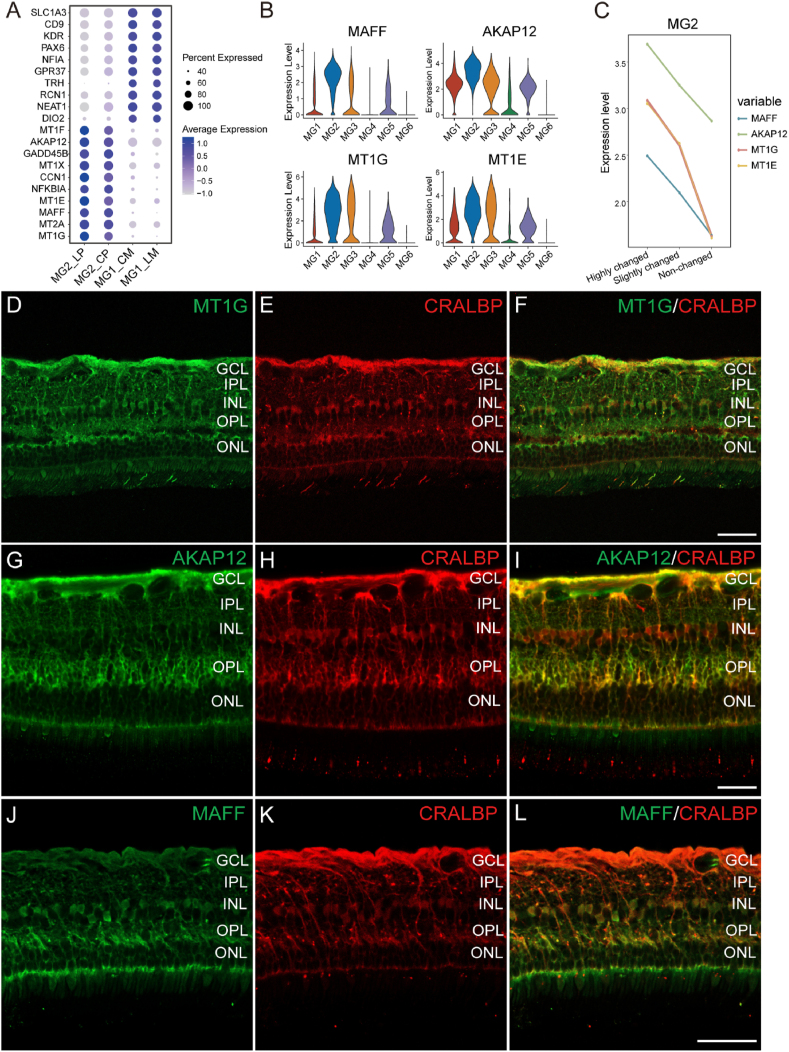
**Genes involved in the response to light stress in periphery-dominant Müller glia and immunofluorescent (IF) staining of MT1G, AKAP12 and MAFF in human peripheral retina**. **A**. Dot plot illustrating the top ten DEGs between MG1 and MG2 cells in response to light stress. **B**. Violin plot showing the expression levels of *MAFF, AKAP12, MT1G* and *MT1E* in subtypes of Müller glia. **C**. Line chart showing the average expression levels of *MT1G*, *MT1E*, *AKAP12* and *MAFF* in MG2 cells in response to light stress. **D**. IF staining of MT1G (green) in human peripheral retina. **E**. IF staining of CRALBP (red), a Müller glia marker. **F**. Co-localization of MT1G and CRALBP. **G**. IF staining of AKAP12 (green) in human peripheral retina. **H**. IF staining of CRALBP (red). **I**. Co-localization of AKAP12 and CRALBP. **J**. IF staining of MAFF (green) in human peripheral retina. **K**. IF staining of CRALBP (red). **L**. Co-localization of MAFF and CRALBP. Scale bar = 50 μM. GCL: Ganglion Cell Layer; IPL: Inner Plexiform Layer; INL: Inner Nuclear Layer; OPL: Outer Plexiform Layer; ONL: Outer Nuclear Layer.

Given the significantly different responses to light stress observed between MG1 and MG2 cells, we investigated the top target genes that were region-specific and associated with pathological processes (Supplementary Data 5). We selected the top ten genes that exhibited differential expression between MG1 and MG2 with or without light stress (Fig. 4A). Substantial transcriptomic changes were observed in MG2 cells in response to light stress, in contrast to MG1 cells, which had minimal changes. Among the top ten genes highly enriched in MG2, *MT1F*, *AKAP12*, *MT1X*, *CCN1*, *MT1E*, *MAFF*, *MT1G* were upregulated in response to light stress. These findings indicate that Müller glia in the human retina consist of distinct subtypes with unique transcriptome profiles exhibiting differential stress responses. We chose three top DEGs, *MT1G*, *AKAP12* and *MAFF*, which were particularly abundant in MG2 (Fig. 4B) and were highly responsive to light stress (Fig. 4C, highly changed) for further study. To determine whether regional differences in these top gene expression were due to intrinsic differences between Müller glia rather than differences in cell numbers, we normalised the expression of MT1G, AKAP12 and MAFF to glial structural marker (RLBP1) (Supplementary Table 3). Even after normalization, these stress-response genes remained more highly expressed in peripheral Müller glia than in macular Müller glia, both at baseline and after light stress, indicating a true per-cell difference in stress responsiveness.

Protein expression patterns of the three top DEGs, *MT1G*, *AKAP12* and *MAFF*, in the human retina, were studied with immunofluorescent staining. We found that MT1G primarily co-localised with cellular retinaldehyde-binding protein (CRALBP), a Müller glia-specific marker (Fig. 4D–F). AKAP12 staining co-localised with CRALBP in the retinal nerve fibre layer, outer plexiform layer and outer limiting membrane of the peripheral retina (Fig. 4G–I). We observed MAFF staining in cell bodies of Müller glia and their processes in outer plexiform layer and outer limiting membrane. (Fig. 4J–L). We validated the protein expression of *MT1G*, *AKAP12* and *MAFF* using freshly obtained retinal tissue punches collected from different locations across the human retina (Supplementary Fig. 6). The protein expression of these genes was consistent with the distinct expression patterns identified in our RNAseq analysis, with lower expression levels observed in the macula than that in the peripheral retina.

### 2.6 Functional validation of *MT1G*, *AKAP12* and *MAFF* in Müller glia in response to light stress

**Fig. 5:**
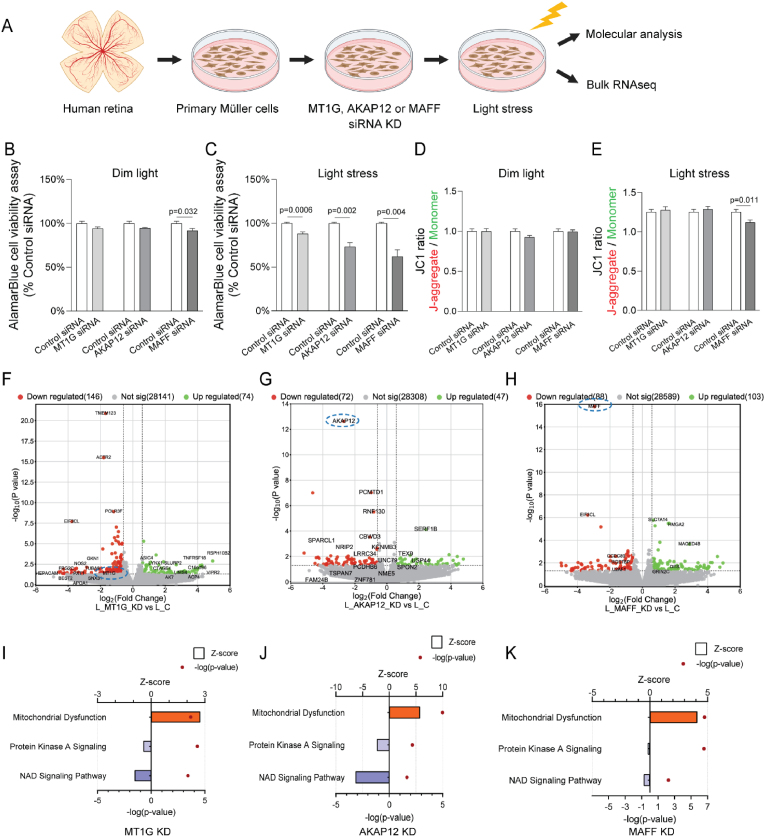
**Knockdown of *MT1G*, *AKAP12* and *MAFF* in human primary Müller glia. A.***MT1G*, *AKAP12* and *MAFF* siRNA knockdown in primary Müller glia exposed to light stress. **B** & **C**. AlamarBlue cell viability assay on primary Müller glia with or without *MT1G*, *AKAP12* and *MAFF* siRNA treatment in response to light stress. n = 6 biological replicates per group. Statistical analysis was performed using Welch's *t*-test (two-sided) between the control group (used consistently across all pairwise comparisons) and the respective knockdown groups. Data are presented as means ± standard error of the mean (SEM). **D** & **E**. JC1 assay on primary Müller glia with or without *MT1G*, *AKAP12* and *MAFF* siRNA knockdown in response to light stress. n = 8 biological replicates per group. Statistical analysis was performed using Welch's *t*-test (two-sided) between the control group (used consistently across all pairwise comparisons) and the respective knockdown groups. **F**–**H**. Volcano plots of differential gene expression (p < 0.05, FC > 1.5) in *MT1G*, *AKAP12* and *MAFF* siRNA knockdown vs. control groups. **I**–**K**. IPA of differential gene expression in human primary Müller glia with *MT1G*, *AKAP12* and *MAFF* siRNA knockdown compared to the control group in response to light stress.

We knocked down *MT1G*, *AKAP12* and *MAFF* in primary human Müller glia with siRNA and exposed them to light stress (Fig. 5A). Knockdown of *MAFF* reduced cell viability by 5–10 % compared with control siRNA treated cells under dim light (Fig. 5B). Under light stress, by contrast, the viability of Müller glia in which *MT1G*, *AKAP12* or *MAFF* had been knocked down decreased by 10–30 % compared with the control (Fig. 5C).

We also evaluated the mitochondrial membrane potential of primary Müller glia using JC-1 staining. The JC-1 ratio (J-aggregate/Monomer) was not significantly different between the control and *MT1G*, *AKAP12* and *MAFF* knockdown groups under dim light (Fig. 5D). However, the JC-1 ratio of the *MAFF* knockdown group was significantly lower than that of the control group under light stress (Fig. 5E). These findings suggest that *MAFF* may play an important role in the Müller glia stress response.

To gain further insights into the function of these genes, we performed bulk RNA sequencing in Müller glia with *MT1G*, *AKAP12* and *MAFF* siRNA treatment, with or without light stress (Supplementary Data 6). The mRNA levels of *AKAP12*, *MAFF* and *MT1G* were significantly reduced, as shown in the volcano plots (Fig. 5F–H, blue circles). Heatmaps of gene expressions between *AKAP12*, *MAFF*, *MT1G* siRNA-treatment groups and the control group displayed distinct clustering of individual samples within the same group (Supplementary Fig. 7).

Ingenuity Pathway Analysis (IPA) analysis revealed several significant differences in canonical pathways that were impacted by *MT1G*, *AKAP12* or *MAFF* knockdown. The commonly altered pathways included mitochondrial dysfunction, protein kinase A signalling and NAD signalling (Fig. 5I–K). The transcription level changes suggested that pathways associated with mitochondrial dysfunction were activated in all three target gene knockdown groups, while pathways related to protein kinase A and NAD signalling pathways could have been inhibited.

### 2.7 Dysregulation of MT1G, AKAP12 and MAFF in human diseased retinas

**Fig. 6:**
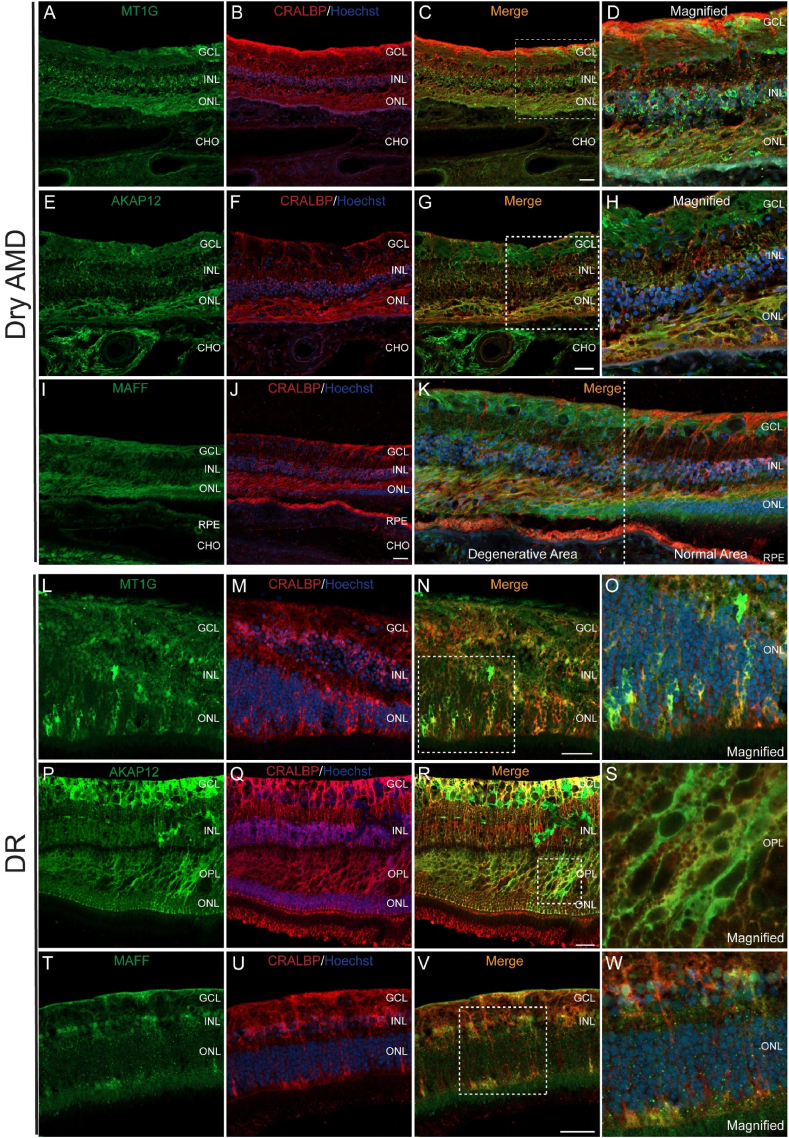
**Dysregulation of *MT1G, AKAP12 and MAFF* in human retinas with dry AMD and DR.** **A-D**. IF staining of MT1G (green) and CRALBP (red) on the donor retina with dry AMD. **A**. IF staining of MT1G. **B**. IF staining of CRALBP and Hoechst (blue). **C.** Co-localization of MT1G and CRALBP. **D**. Magnified image of the dotted box in **C**. **E-H**. IF staining of AKAP12 (green) and CRALBP (red) on the donor retina with dry AMD. **E**. IF staining of AKAP12. **F**. IF staining of CRALBP and Hoechst (blue). **G**. Co-localization of AKAP12 and CRALBP. **H**. Magnified image of the dotted box in **G**. **I–K**. IF staining of MAFF (green) and CRALBP (red) on the donor retina with dry AMD. **I**. IF staining of MAFF. **J**. IF staining of CRALBP and Hoechst (blue). **K**. Co-localization of MAFF and CRALBP. **L-O**. IF staining of MT1G (green) and CRALBP (red) on the donor retina with DR. **L**. IF staining of MT1G. **M**. IF staining of CRALBP and Hoechst (blue). **N**. Co-localization of MT1G and CRALBP. **O**. Magnified image of the dotted box in **N**. **P–S**. IF staining of AKAP12 (green) and CRALBP (red) on the donor retina with DR. **P**. IF staining of AKAP12. **Q**. IF staining of CRALBP and Hoechst (blue). **R**. Co-localization of AKAP12 and CRALBP. **S**. Magnified image of the dotted box in **R**. **T-W**. IF staining of MAFF (green) and CRALBP (red) on the donor retina with DR. **T**. IF staining of MAFF. **U**. IF staining of CRALBP and Hoechst (blue). **V**. Co-localization of MAFF and CRALBP. **W**. Magnified image of the dotted box in **V**. Scale bar = 50 μM.

We analysed the expression of MT1G, AKAP12 and MAFF in postmortem human retinas with documented age-related macular degeneration (AMD) with geographic atrophy and diabetic retinopathy (DR) (Supplementary Fig. 8A–D). Geographic atrophy in the retinas with AMD were characterised by loss of the photoreceptors and retinal pigment epithelium with extensive Müller glia filaments (fibrotic tissue) filling the space (Fig. 6B and F and **6J**).

We observed abnormal expression of MT1G, AKAP12 and MAFF (green, Fig. 6A, E and 6I) in these degenerative areas. MT1G staining was still primarily co-localized with the Müller glia marker CRALBP (Fig. 6B and C). Notably, MT1G exhibited unusually strong staining in the outer nuclear layer (ONL), particularly in the areas of geographic atrophy in the retina with AMD (Fig. 6D). AKAP12 was still mainly expressed within Müller glia, as evidenced by its co-localization with CRALBP staining (Fig. 6E–H), with unexpectedly strong staining in the ONL. The immunostaining of MAFF also showed abnormalities. Although MAFF was still co-localized with CRALBP, it appeared upregulated in the degenerated areas, especially at the margins (Fig. 6K, left). In contrast, the expression level was relatively low in more regular areas (Fig. 6K, right).

The retina with DR exhibited significant disruption (Fig. 6M and Q) or thinning (Fig. 6U) of the inner nuclear layer (INL). We observed unusual, patchy upregulation of all three target proteins—MT1G, AKAP12 and MAFF—within the Müller glia of the DR retina (Fig. 6L, N, 6P, 6R, 6T and 6V). The patchy activation of MT1G was primarily localised to the ONL (Fig. 6O). AKAP12 was patchily activated in the outer plexiform layer (Fig. 6S). MT1G and AKAP12 were also elevated in the walls of intraretinal cysts (Supplementary Fig. 8E–H), though this may be partly due to condensation of Müller glia in these regions. The patchy upregulation of MAFF staining was predominantly observed in the cell bodies of Müller glia within the INL and processes in the ONL (Fig. 6W).

### 2.8 Dysregulation of MT1G, AKAP12 and MAFF in retinal disease mouse model

**Fig. 7:**
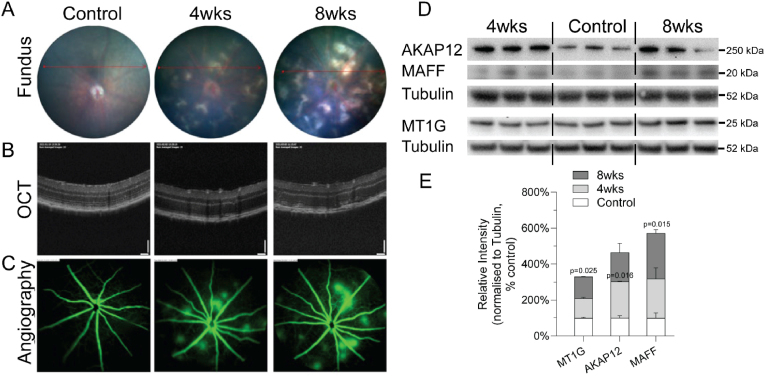
**Dysregulation of *MT1G, AKAP12* and *MAFF* in JR55558 mice mimicking subretinal neovascularisation. A**. fundus photos (**A**), OCT (**B**) and FFA (**C**) images of the C57BL/6J control and JR5558 mice at 4 and 8 weeks of age. **D & E**. Images of representative protein bands and the corresponding densitometry analysis of Western Blot against MT1G, AKAP12 and MAFF in the retinas of both control and JR5558 mice at the ages of 4 and 8 weeks of age. n = 3 mouse retinas per group. Statistical analysis was performed using Welch's *t*-test (two-sided) between the control and the 4 weeks or 8 weeks old groups, respectively.

We examined the protein expression of *MT1G, AKAP12* and *MAFF* in JR5558 mice that spontaneously develop bilateral subretinal neovascularisation [15,16]. Subretinal lesions appeared around 4 weeks and became established at 8 weeks, as confirmed by colour fundus photography, optical coherence tomography and fundus fluorescein angiography (Fig. 7A–C). We compared the protein expression of *MT1G, AKAP12* and *MAFF* in the JR5558 mouse retina with the C57BL/6 control retina. We found that AKAP12 was upregulated at both 4 weeks and 8 weeks of age in the JR5558 mouse retina compared with the control retina (129 % vs 100 %, 169 % vs 100 %) (Fig. 7 D&E). The protein expression of *MAFF* and *MT1G* was significantly greater at 8 weeks of age in the JR5558 mouse retina than in the control (217 % vs 100 %, 121 % vs 100 %, respectively). These results show that the stress-responsive genes *MT1G, AKAP12* and *MAFF* are activated in subretinal neovascularisation, which is a common vision-threatening mechanism in retinal disease.

## 3 Discussion

Though Müller glia are found in both the macula and the peripheral retina, disparities exist in their distribution, density, morphology and molecular functions across these regions [6]. This study examined the redox stress responses of Müller glia in both areas utilising scRNAseq on human macular and peripheral retinal explants. We found that macular Müller glia exhibited a blunted transcriptional response to oxidative stress compared to their peripheral counterparts. Analysis of the top three genes most abundantly expressed in peripheral Müller glia—*MT1*, *AKAP12* and *MAFF*, which are involved in redox regulation—revealed a crucial role in cellular survival under oxidative stress. These findings suggest that the regional differences in redox homeostasis may contribute to the macular region's heightened susceptibility to oxidative damage and degenerative diseases such as AMD, macular edema and MacTel.

Although cones are densely concentrated in the foveal center, their overall contribution to total cell numbers in a 5 mm macular punch is relatively small due to the high abundance of rods in the parafoveal and perifoveal macula. Rods outnumber cones by approximately 4:1 at 0.7 mm from the foveal center and even more so in the broader macular region [17,18]. This is why the relative proportion of cones captured by scRNA-seq differed only modestly between macula and periphery (∼2.3 % vs. ∼1.7 %). In contrast, retinal ganglion cells and Müller glia—both topographically enriched in the macula—showed more pronounced regional proportional differences. Notably, macroglial cells (Müller glia and retinal astrocytes) constituted a larger fraction of cells in the macular samples than in the peripheral samples, reflecting the dense glial support network of the central retina. The greater abundance of Müller glia in the macula may provide enhanced support to macular neurons under normal conditions, but this numerical advantage might not extend to better stress resilience if macular Müller glia are inherently less stress-responsive (as our data suggest for certain oxidative stress genes).

Metallothioneins (MTs) are a group of low molecular weight, cysteine-rich proteins that play critical roles in various biological processes, including heavy metal detoxification [19], oxidative stress protection [20] and apoptosis regulation [21,22]. Among them, MT1 functions as a key antioxidant, scavenging ROS and maintaining cellular redox balance. MT1 has been implicated in neuroprotection against age-related oxidative stress [23,24], as it is associated with aging and endoplasmic reticulum (ER) stress, particularly in defending against nitric oxide (NO)-induced damage. Given that NO-induced oxidative stress contributes to retinal diseases such as DR, glaucoma and AMD, the reduced expression of MT1 in macular Müller glia may contribute to the macula's vulnerability to oxidative insults [25].

AKAP12 (also known as SSeCKS/Gravin) is a large scaffolding protein that tethers multiple signalling enzymes—including PKA, PKC, Src-family kinases and cyclins—to precise sub-cellular domains, thereby controlling the intensity and duration of stress-responsive pathways in many tissues [26]. In the central nervous system, of which the retina is a part, AKAP12 expression in astrocytes and endothelial cells promotes both angiogenic pruning and tight-junction maturation: loss of AKAP12 causes excess VEGF, reduced ZO-1 and pathological vascular leak, whereas its over-expression increases the anti-permeability factor Angiopoietin-1 and strengthens barrier integrity [[27], [28], [29], [30]]. By analogy, Müller glia—the functional retinal homologues of astrocytes—may use AKAP12 to coordinate neurovascular crosstalk; its markedly higher basal expression and stronger light-inducibility in peripheral versus macular Müller cells suggest that peripheral glia possess a more robust AKAP12-centred toolkit for maintaining barrier and tissue homeostasis under stress. Although AKAP12 itself has not been directly linked to redox control, other family members illustrate how an AKAP platform can modulate oxidative resilience: mitochondrial AKAP1 (D-AKAP1) anchors PKA to the outer mitochondrial membrane, limits ROS generation and preserves membrane potential after hypoxia [31]. We speculate that retinal AKAP12 may organise analogous kinase/phosphatase assemblies at Müller-glial end-feet or mitochondria, helping peripheral Müller cells buffer oxidative or inflammatory insults more effectively than their macular counterparts. Consistent with a stress-adaptive role, we found that AKAP12 protein was focally up-regulated in Müller processes within geographic atrophy and diabetic-retinopathy specimens (Fig. 6), and Ingenuity Pathway Analysis of AKAP12-knockdown Müller cells predicted activation of mitochondrial-dysfunction pathways. Together, these data position AKAP12 as a candidate coordinator of neurovascular and metabolic defence mechanisms whose regional expression bias could contribute to the macula's relative vulnerability.

MAFF protein belongs to the small Maf family of proteins that are known to form heterodimers with Nrf2, a master regulator of antioxidant response elements (AREs), thereby modulating transcriptional activation of oxidative stress defense genes [32,33]. There is limited information regarding the specific role of MAFF proteins in human diseases, but they have been implicated in various pathological conditions such as cancer, diabetes, immune system disorders and neurodegenerative diseases such as Alzheimer's disease and Parkinson's disease [[34], [35], [36], [37], [38]]. In the context of retinal biology, Nrf2-Maf interactions play a critical role in cellular defense against oxidative damage, particularly in Müller glia, which are key regulators of retinal redox homeostasis [33]. The role of MAFF in Müller glia has not been previously investigated. However, its elevated expression in Müller glia, especially at the margins around the degenerative area in the AMD, suggests it may modulate the Nrf2-related stress response in these cells. Further study is warranted to elucidate the roles of MAFF in Müller glia and other biological processes; this is beyond the scope of the current study.

MT1G, AKAP12 and MAFF are much more strongly expressed in Müller glia of the peripheral retina than of the macula. siRNA knockdown of these genes in human primary Müller glia disrupted multiple stress response signalling pathways, including mitochondrial function and the protein kinase A and NAD signalling pathways. A better understanding of how the macula responds to stress when it only expresses stress response genes weakly and non-inducibly may pinpoint new treatment targets for macular diseases.

The intrinsic deficiency of macular Müller glia that we have observed may be a result of their environment evolving to provide additional external shields against light-derived oxidative load. The foveal/macular region is protected by (i) densely pigmented RPE cells that absorb short-wavelength photons and recycle chromophores [39] and (ii) a high concentration of macular xanthophyll pigments (lutein, zeaxanthin) that filter blue light and quench singlet oxygen [40]. Living under this dual photoprotection, macular Müller cells may not need to maintain the same basal repertoire of redox-response genes that peripheral Müller cells deploy. Our data therefore suggest a context-dependent vulnerability: compromise of RPE function or macular pigment, such as in early AMD, leaves the macula to rely on an internal glial defence system that is comparatively under-expressed, accelerating local damage. This hypothesis will require direct testing.

Notably, in diseased human maculas (AMD and diabetic retinopathy samples) we observed elevated MT1G, AKAP12, and MAFF in Müller glia at the margins (Fig. 6), suggesting that macular Müller cells can activate these pathways, but perhaps only in the face of significant stress or damage. Thus, the presence of these proteins in the macula is more likely a marker of ongoing stress or injury than a pre-existing protective factor. In the JR5558 mice, the higher expression of these genes in the context of neovascular stress did not prevent retinal pathology; instead, it accompanied it (Fig. 7). The macular Müller glia's lack of these defenses at baseline might allow damage to initiate more readily, but once damage is underway, those cells do mount a response (hence the genes turn on in disease). However, by that stage, it might be insufficient to halt disease progression. Meanwhile, having these genes expressed from the start in the peripheral Müller glia might delay or reduce the impact of stress, thereby conferring greater resilience. This may explain the spatial differences in retinal disease patterns, albeit one that likely works in concert with many other factors.

AMD is widely accepted to originate with RPE dysfunction [41]; our findings add a complementary layer by showing that macular Müller glia express weaker baseline antioxidant programmes than peripheral Müller glia. When the RPE barrier fails, this second-line defence may become decisive: inferior Müller-cell support in the macula could accelerate photoreceptor loss, whereas stronger peripheral Müller-cell defences help limit damage. Thus, RPE pathology and regional Müller-cell heterogeneity together shape AMD topography, and therapies targeting the RPE might be enhanced by approaches that boost Müller-cell redox capacity.

Studying the macula is challenging because laboratory animals do not have maculas apart from non-human primates, which raises ethical issues. We addressed this by using *postmortem* human retinal/macular explants to investigate the stress response of the human macula. The data derived from this model system complement other models utilized in retina research, such as mice and organoids. One of the primary advantages of using human retinal explants is the direct examination of gene expression and morphology of the human macula in its entirety. Inherent limitations of this system include the lack of a functional vascular network and interaction with the RPE. The RPE normally protects the retina by absorbing blue light and quenching ROS. Consequently, the responses observed in our explants may mimic those of an acutely detached or RPE-deprived retina rather than a fully intact eye in *vivo*. This would not, however, be expected to explain the differences we observed between macular and peripheral retinal explants, which were both cultured without the RPE. These disadvantages could potentially be addressed with perfusion and co-culture systems. The use of postmortem tissue may raise concerns regarding viability and metabolic activity, but there is growing evidence to support the functionality of cultured human retinal explants despite *postmortem* delay [42]. We have previously reported that the metabolic features of human retinal explants at least as good as those of mouse retinas and retinal organoids [43].

Our use of acute intense light exposure as a model for the chronic stress associated with aging may not perfectly reflect the true *in vivo* response of the human macula and peripheral retina. Despite this, our method is a valuable starting point for investigating the stress response of the macula. Light exposure is a well-established inducer of oxidative stress in retinal cells, leading to increased ROS production and mitochondrial dysfunction. While acute light-induced stress may not fully replicate the progressive oxidative damage observed in aging and disease, it provides important insights into how macular and peripheral Müller glia differentially respond to oxidative insults. It also sets the stage for future, in-depth research on neurotoxins, such as deoxy-sphinganine (unpublished data), that significantly influence the pathogenesis of macular telangiectasia type 2 [44] and it helps us begin to unravel the distinct ways the macula responds to stress. Notably, acute light exposure did not lead to significant upregulation of GFAP in Müller glia (a hallmark of gliosis), suggesting that the observed oxidative stress gene changes occur in the absence of reactive Müller cell gliosis. Exposure to light stress induced distinct patterns of cellular responses in the peripheral retina and the macula. Our single-cell RNA sequencing analysis revealed that both Müller glia and rods exhibited significant differential gene expression in the peripheral retina. Cones, horizontal cells and bipolar cells also showed differential gene expression, though to a lesser extent. We focused on the Müller glia response to light stress primarily because Müller glia presented two subpopulations with distinct transcriptomic profiles—one predominantly in the macula and the other in the peripheral retina. Müller glia are among the first responders to retinal stress, playing crucial roles in maintaining retinal homeostasis and responding to injury [4]. Additionally, it is well established that the phototransduction cascade in photoreceptors is one primary source of oxidative stress and cell death [10]. However, intense light exposure in the retina can induce damage through several routes in addition to oxidative stress, such as direct photochemical injury and inflammation, and our findings likely reflect a combination of these effects. Müller glial cells also play a crucial role in the cone Müller visual cycle, a specialized pathway that supports the rapid regeneration of visual pigments in cone photoreceptors, which are essential for colour vision and high-acuity vision in bright light [45,46]. Therefore, the transcriptomic differences observed in Müller glia between the periphery and the macula may also be influenced by the varying densities of rods/cones in these regions, with a lower proportion of rods present in the macula.

Amacrine cells, which play a crucial role in modulating signals between bipolar and ganglion cells, may have evolved to meet the high visual demands of the macular region. This specialization could make them more sensitive to changes in light conditions, ensuring accurate and rapid visual perception. Although the alteration score for amacrine cells is highest in the macula, the number of amacrine cells detected in our dataset is relatively low (1071 out of 77,405 cells, ∼1.4 %). This limitation is a recognized challenge of the scRNAseq technique, where there is less confidence in the findings from cell subpopulations that are only present in small numbers. Consequently, while we acknowledge the potential importance of the observed changes in amacrine cells, the current dataset does not provide sufficient resolution to draw definitive conclusions.

In conclusion, we observed that macular Müller glia exhibit a subdued response to stress, possibly due to the higher functional demands placed on them in the macular region. These cells express low levels of several key stress-response genes that significantly contribute to the resilience of peripheral Müller glia under stress. Future investigations are needed to clarify further the molecular mechanisms underlying the distinct stress response characteristics of macular Müller glia. Understanding how regional differences in redox homeostasis influence macular vulnerability may provide insights into novel therapeutic strategies for oxidative stress-related retinal diseases, including AMD and MacTel. Our findings indicate that this reduced stress response in macular Müller glia could offer an evolutionary advantage by allowing these cells to sustain their normal functions even under acute stress. However, this adaptation may come at the cost of increased susceptibility to chronic oxidative damage, potentially accelerating degenerative processes in the macula.

## 4 Materials and methods

### 4.1 Tissue collection and retinal dissection

The use of donated human eyes was approved by the Human Research Ethics Committee of the University of Sydney (Protocol Numbers: 2016/282) and Sydney Local Health District Ethics Review Committee (2020/ETH03339). Donors had no known eye diseases. Retinal dissection was conducted as previously described [47]. In brief, donor eyes were acquired from the New South Wales Tissue Bank after the corneas had been removed for transplantation. The donor tissues were preserved in CO_2_-independent medium at 4 °C until they were dissected. For dissection, the eye was placed on a Petri dish and the iris, lens and vitreous were excised from the eyecup. The neural retina was then carefully detached from the underlying retinal pigment epithelium and transferred to a fresh Petri dish. Circular retinal tissues with a diameter of 5 mm were collected from the macula or mid-peripheral region of the superior retina by punch biopsy (#BP-50F, Kai Medical) and processed for downstream assays. The mid-peripheral region was identified as the midpoint between the fovea and ora serrata. 2 mm-diameter circular retinal samples extending from the fovea to the superior and inferior peripheral neural retina (Supplementary Fig. 6A) were collected and extracted for protein analysis. One postmortem eye from a donor with diabetic retinopathy (DR) was used to validate the expression of target genes in the diseased retina. Detailed fundus photography was conducted to document characteristics and features present within the diseased retina.

### 4.2 Light and high glucose stress models on retinal explants and human primary Müller glia

We employed a custom-built light exposure system situated within the tissue culture incubator to induce light stress in retinal explants and human primary Müller glia (Supplementary Fig. 1). Light stress was accomplished by subjecting explants to intense light at 32k lux, while the control group was exposed to 5 lux light for a continuous period of 4 h. The light source was positioned at a distance of 10 cm from the samples in both the experimental and control groups to ensure uniform light exposure. Retinal explant tissues were placed on 12-well plate transwell inserts (#3460, Corning) with neurobasal medium (#21103049, Gibco) supplemented with 1 % B27 (#17504044, Gibco), 1 % N2 (#17502048, Gibco), 1 % fetal bovine serum (FBS, #F9423, Sigma‐Aldrich), 1 % GlutaMax (#35050, Gibco), 1 %ITS (#51500056, Gibco) and 1 % penicillin-streptomycin (P/S, #P4333, Sigma‐Aldrich). Macular and peripheral neural retinal explants were exposed to light for 4 h (Fig. 1A). Three days after siRNA transfection, human primary Müller glia in 12-well (#3513, Corning) or 96-well plates (#3340, Corning) were exposed to intense (32k lux) or dim (5 lux) light. Macular and peripheral retinal explants were cultured in either normal glucose (5 mM glucose, left eye) or high glucose (25 mM, right eye of the same donor) on 12-well transwell inserts with DMEM (#10569, Gibco) supplemented with 1 % B27, 1 % N2, 1 % FBS and 1 % P/S for 24 h.

### 4.3 Single cell dissociation and cDNA library construction

Macular and peripheral explants were dissociated into single cells following light or high glucose treatment as depicted in Fig. 1A. Single-cell dissociation was achieved using the Papain Dissociation System (Worthington Biochem, NJ, USA) according to the manufacturer's protocol. Briefly, the entire retinal explant was immersed in papain solution for 30 min at 37 °C, followed by dissociation through pipetting with a glass pipette. The single-cell suspension was combined with barcoded gel beads in a microfluidic chip and run on the Chromium Controller (10X Genomics) to create gel bead-in-emulsion (GEMs) droplets (Chromium Next GEM Single Cell 3ʹ Kit v3.1, 16 rxns PN-1000268, 10 × Genomics). The GEMs were incubated, enabling mRNA reverse transcription into cDNA. The barcodes from the gel beads were incorporated into the cDNA molecules by uniquely tagging the cDNA originating from each individual cell. The barcoded cDNA was then amplified using PCR to construct a library. The libraries were sequenced on an Illumina NovaSeq 6000 platform.

### 4.4 Single cell RNA sequencing data analysis

The raw sequencing data was processed by the Cell Ranger software (v3.0.2, 10x Genomics). A human reference genome (GRCh38) was used to accurately map the sequencing readouts and quantify gene expression. We then applied Seurat (v4.1.1) [48] to perform the pre-processing and downstream analysis of the single-cell gene expression data. Cells with an expression of unique feature counts over 6000 (as for the high glucose treated group, this parameter was 10,000) or less than 200, a mitochondrial gene ratio over 15 % and a hemoglobin gene ratio over 5 % were filtered out. DoubletFinder [49] (v2.0.3) was used to detect and remove the doublets as to the following criteria: pN = 0.25, pK = 0.09, PCs = 1:20, sct = TRUE and reuse.pANN = FALSE. After the above quality control, we normalised and log-transformed the featured expression measurements for each cell. 2000 highly variable features were chosen for downstream data scaling. The integration of multiple single-cell datasets from different biological contexts was implemented by SeuratWrappers (v 0.3.0) with LIGER algorithm. It depends on integrative non-negative matrix factorisation to delineate shared and dataset-specific factors of cell identity [50]. Then the “FindNeighbors” and “FindClusters” functions were applied for unsupervised clustering. For visualization, UMAP (Uniform Manifold Approximation and Projection) was used to reduce the dimensionality. We further annotated each cell cluster based on specific marker gene expression. The differentially expressed genes were calculated by the “FindMarkers” function with specific settings (only.pos = FALSE, min.pct = 0.2, logfc.threshold = 0.2)

### 4.5 Calculating the alternation score of different cell types

To quantify the change in cellular response to light, we designed the Alternation (AC) Score. The first step of selecting the signature genes was conducted as a previously published method that characterized the MI contribution [11]. However, we have modified the second step to calculate both the quantity and expression level change of signature genes during the light stress process. Here, we defined the new ${AC}_{score}$ as follows:

(1) $${AC}_{score\left(j\right)}=SUM\;\log\;2\left({FC}_{\exp\left(i\right)}\cdot {FC}_{prop\left(i\right)}+1\right)$$

In which ${FC}_{\exp\left(i\right)}$ refers to the expression fold change of the ith signature gene in the jth cell type between the light stress and control groups, ${FC}_{prop\left(i\right)}$ is the proportion fold change of the cells that express the ith signature gene between the light stress and control groups in the jth cell type.

### 4.6 Light stress-associated relative likelihood analysis

MELD algorithm implemented in Python (v.3.8.16) was employed to calculate the continuous light stress-associated relative likelihood of each cell across the entire single-cell dataset [51]. In brief, the density of each sample (replicates and conditions) was estimated on a MELD graph built over the cellular transcriptomic state space (beta = 67, knn = 7). We then compared the density estimates between strong and dim light conditions within each replicate. The resulting relative likelihoods across replicates were normalised to quantify the probabilities of a given cell would be observed in the light stress condition. Cut-offs of values less than 0.4, between 0.4 and 0.6 and greater than 0.6 were used to determine whether a cell was non-changed, slightly changed, or highly changed, respectively.

### 4.7 Single-cell trajectory analysis

We utilized the 10 × velocyto pipeline on the filtered cell ranger-generated BAM files to determine the number of spliced and unspliced reads for each sample. Then, we employed the dynamical model of scVelo v.0.2.3 [52] for single-cell RNA velocity inference. We obtained a comprehensive view of RNA velocity across all genes and cells by visualizing a vector field on top of the 2D dimensional reduction plot. We enhanced our interpretation by leveraging partition-based graph abstractions (PAGA) to ascertain the directionality between cells.

### 4.8 Gene Ontology analysis

Gene Ontology (GO) analysis was conducted using the web-based resource, the Generic Gene Ontology Term Finder [53], accessible at https://go.princeton.edu/cgi-bin/GOTermFinder. Highly expressed genes in macula-dominant Müller glia (*m*-dMG) were defined as “m-HXs”. Highly expressed genes in peripheral retina-dominant Müller glia (*p*-dMG) were defined as “p-HXs”. Light-induced changes were defined as “LiC”. GO analyses were performed on the *m*-HXs and *p*-HXs (fold change >1.5) with (adjusted p value < 0.05) or without LiC (adjusted p value > 0.05).

### 4.9 Ingenuity Pathways Analysis

QIAGEN Ingenuity Pathway Analysis (IPA) was performed to identify significant canonical pathways and predict which pathways were activated or inhibited between control and MT1G, MAFF and AKAP12 siRNA treatment groups. Upregulated and downregulated genes were imported into the IPA software and subjected to core analysis to identify canonical pathways.

### 4.10 Vibratome sectioning, immunofluorescent staining and imaging

Human retinal tissue from the macula and mid-periphery was obtained using a 5 mm-diameter biopsy punch after separating the neural retina from the RPE-choroid-sclera eyecup. Tissues were fixed in 4 % paraformaldehyde for 1 h and then washed with 1 × phosphate-buffered saline (PBS). Retinal tissues were embedded in 3 % low-melting point agarose (#50100, Lonza) within a plastic cubic mould. The tissue blocks were secured onto the specimen plate of a vibratome (#VT1200S, Leica). Serial tissue sections, 100 μm thick, were generated through vibrating sectioning. The tissue slices were maintained in cold 1 × PBS until further use. Retinal slices were blocked overnight in blocking buffer (5 % normal donkey serum and 0.5 % Triton-X100 in PBS) in a 48-well plate (#3548, Corning). Primary antibodies (Table 1) were diluted in 1 % normal donkey serum and 0.5 % Triton-X100 in PBS. Tissues were incubated in primary antibody for consecutive 5 days, then washed three times with PBS and placed in a diluted secondary antibody (1 % normal donkey serum and 1 % Triton-X100 in PBS) for two days. Tissues were then washed 3 times with PBS. Nuclei were stained with Hoechst 33342 for 30 min in the dark. Slices were mounted onto polysine-coated slides (Menzel Glaser, Germany) with secure-seal spacers (ThermoFisher Scientific, MA, USA) using VECTASHIELD mounting medium (#H-1000, Vector Laboratories). Confocal images were taken using a confocal microscope and imaging system (Zeiss LSM700). Zen Software (blue edition) from Zeiss was used to analyse the images.

**Table 1:** List of antibodies used in the study.

Antibodies	Source	Catalogue NO.	Host	Dilutions	
				WB IF	
α/β-Tubulin	Cell signalling	#2148	Rabbit	1:1000	N/A
CRALBP	Abcam	AB15051	Mouse	N/A	1:500
MAFF	Abcam	AB183859	Rabbit	1:1000	N/A
MAFF	Novus	NBP2-56792	Rabbit	N/A	1:200
AKAP12	Sigma	HPA006344	Rabbit	1:1000	1:200
MT1G	Abcam	AB193329	Rabbit	1:1000	1:200
CRALBP	Abcam	AB1243664	Rabbit	N/A	1:500
Anti-rabbit-488	Invitrogen	A21206	Donkey	N/A	1:1000
Anti-mouse-594	Invitrogen	A21203	Donkey	N/A	1:1000
Hoechst 33342	Thermo Fisher	H3569	N/A	N/A	1 μg/mL
Anti-rabbit IgG	Cell signalling	#7074	Goat	1:5000	N/A
Anti-mouse IgG	Cell signalling	#7076	Horse	1:5000	N/A

### 4.11 Cryosectioning and immunofluorescent staining

The human AMD eyes were fixed in 4 % paraformaldehyde (PFA) prepared in PBS buffer for 24 h. After fixation, the macular retina, choroid and sclera were dissected together and transferred to 20 % sucrose in PBS for cryoprotection, incubating overnight at 4 °C. The tissue was then embedded in optimal cutting temperature compound and sectioned at a thickness of 16 μm onto Superfrost glass slides. For immunofluorescent staining, tissue sections were blocked in a solution containing 5 % donkey serum and 1 % Triton X-100 in PBS for 1 h to prevent non-specific binding. The primary antibody was diluted in PBS containing 1 % donkey serum and 1 % Triton X-100 and incubated with the sections for 48 h at 4 °C. Following primary antibody incubation, the sections were washed and then incubated with the secondary antibody diluted in PBS containing 1 % donkey serum for 4 h at room temperature. Nuclei were stained with Hoechst33342 for 5 min at room temperature. After the staining, the tissue sections were washed three times with PBS and mounted using VECTASHIELD Antifade Mounting Medium (Vector Laboratories, Burlingame, CA, H-1000) before placing coverslips for imaging.

### 4.12 Western Blot

Samples of 2 mm diameter retinal punches were collected from donor eyes (Supplementary Fig. 1A) and homogenized on ice using radioimmunoprecipitation assay (RIPA) buffer (#900000064244, Merck) supplied with phosphatase and protease inhibitor (1:100, #5872S, Cell Signaling Technologies). Human primary Müller glia were lysed in RIPA buffer with phosphatase and protease inhibitor in a 12-well plate. Tissue and cell lysate were centrifuged at 12,000 g at 4 °C for 15 min. Supernatants were collected and aliquoted. Protein concentration was determined using a BCA assay kit (#QPBCA, Sigma‐Aldrich). Protein samples were mixed with DTT (#D9779-10G, Sigma) and loading sample buffer (#NP0007, Invitrogen), heated at 75 °C for 10 min and then cooled on ice for 2 min. Samples were loaded onto the 3–8 % Tris acetate gel (#EA03785BOX, Invitrogen) for AKAP12 evaluation and electrophoresed at 150 V, 4 °C for 60 min. Samples were loaded onto 10–20 % Tricine gels (EC66255BOX, Invitrogen) for the evaluation of MAFF and MT1G and were electrophoresed at 125 V, 4 °C for 90 min. Proteins were transferred onto PVDF membranes (#IEVH85R, Millipore, USA) using the wet transfer system (#1703930, BioRad, USA), following the manufacturer's instructions. PVDF membranes were blocked with 5 % bovine serum albumin (BSA, #A9647-100G, Sigma) in 1 × tris-buffered saline (TBS) for 1 h at room temperature and then incubated with primary antibodies (Table 1) diluted in 1 % BSA in tris-buffered saline with tween 20 (TBST) overnight at 4 °C. Membranes were then washed three times with TBST and incubated with secondary horseradish peroxidase (HRP)-antibodies in 1 % BSA in TBST for 2 h. Membranes were then washed three times with 1 × TBST and twice with 1 × TBS. Protein bands were visualized using Clarity ECL substrate (#170–5061, BioRad) and photographed using the G-Box imaging system (In Vitro Technologies). The specific bands of interest were quantified using the GeneTools image scanning and analysis package. Protein expressions were normalised to α/β tubulin.

### 4.13 Isolation and culture of primary Müller glia from human donor retinas

Human primary Müller glia were cultured and passaged following the method described previously [47]. Human donor eyes (Supplementary Table 4), with corneas removed, were transported in CO_2_-independent medium (#18045088, ThermoFisher Scientific). Retinas were detached from the eyecups, cut into 1 cm^2^ pieces and stored in Dulbecco's modified Eagle medium (DMEM, #10569010, ThermoFisher Scientific) without FBS or growth factors at 4 °C overnight in the dark. The next day, retinal tissue was digested in pre-warmed TrypLE (#12563029, ThermoFisher Scientific) at 37 °C for 60 min, then transferred to a culture dish with complete medium and cut into ∼1 mm^2^ pieces. Retinal pieces were placed into T25 cell culture flasks (#3289, Corning), separated with an angled 18 G needle and pressed firmly onto the flask bottom to enhance attachment. Flasks were incubated vertically for 15 min in the 37 °C incubator, then horizontally with 2 ml complete medium (DMEM+10 % fetal bovine serum +1 % Penicillin-Streptomycin). On day 7, an additional 2 ml of medium was added into the T25 cell culture flask. Routine culture involved medium changes twice a week and after 2–3 weeks, human Müller glia colonies emerged, reaching confluency in another 2–3 weeks. For passaging, cells were rinsed with PBS, digested with TrypLE, centrifuged and resuspended in complete medium, then seeded in new flasks. Subsequent passaging was done at 1:1 initially, progressing to 1:2 or 1:3 ratios after P1, with experiments typically using P3 cells.

#### 4.13.1 siRNA treatment

Human primary Müller glia (P3) at 80 % confluency were transfected with *MT1G* (assay ID: s194623, ThermoFisher Scientific), *AKAP12* (assay ID: s18435, ThermoFisher Scientific), *MAFF* (assay ID: s24370, ThermoFisher Scientific) and control small interfering RNA (siRNA) (assay ID: 4390843, ThermoFisher Scientific) at a concentration of 10 nM using Lipofectamine 3000 (#L3000-008, Invitrogen), following the manufacturer's instructions. Müller glia were treated with siRNA in either 12-well or 96-well plates for three days, reaching 100 % confluency prior to further experiments.

#### 4.13.2 AlamarBlue assay

Human primary Müller glia (P3) were transfected with *AKAP12*, *MT1G*, *MAFF* and control siRNA in a 96-well plate for three days, then starved overnight and exposed to light stress for 4 h. Cells were treated with AlamarBlue reagent (1:10 dilution, #DAL1100, ThermoFisher Scientific) and incubated at 37 °C for 4 h using the AlamarBlue cell viability assay kit. The fluorescence was then read using the microplate reader (Fluostar Omega, BMG Labtech) with the 544 nm excitation wavelength and 590 nm emission wavelength.

##### 4.13.2.1 JC1 assay

Human primary Müller glia (P3) after siRNA treatments were incubated with JC-1 dye (#T3168, Invitrogen) at a concentration of 1.0 μg/mL in DMEM at 37 °C for 30 min to measure the mitochondrial membrane potential. The medium was replaced with Hanks’ balanced salt solution (HBSS) and fluorescence was measured using a plate reader. The fluorescence was detected with the 475 nm excitation wavelength and 530 nm for green emission and 590 nm for red emission. The ratio of red fluorescence divided by green fluorescence of each sample was calculated. All data were normalised to the control siRNA treatment group.

### 4.14 mRNA extraction, bulk RNA sequencing and bioinformatic analysis

Total mRNAs were extracted from human primary Müller glia treated with *AKAP12*, *MT1G*, *MAFF* and control siRNA using the GenEluteTM Single Cell RNA Purification Kit (#RNB300, Sigma-Aldrich), following the manufacturer's instructions. The concentration of mRNA was determined using the Qubit RNA HS Assay Kit (#Q32852, Invitrogen). The extracted mRNA samples were sent to Novogene (Singapore) for bulk RNA sequencing after quality control assessment. The library was prepared and a 150 bp paired-end sequencing strategy was used to sequence the samples. The quality of the library was analysed using the Agilent Bioanalyzer 2100 system.

### 4.15 Mouse eye examinations and retinal tissue collection

Approval for all experimental procedures was obtained from the University of Sydney Animal Ethics Committee, ensuring adherence to ethical standards and the welfare of animals (Project number: 2021/2013). The study utilized JR5558 mice (strain #:005558; RRID:IMSR_JAX:005558) obtained from The Jackson Laboratory for validating the expression of AKAP12, MT1G and MAFF proteins in diseased mouse retinas. C57BL/6J wild-type mice served as the control group.

Mice were housed in individually ventilated cages under specific pathogen-free conditions, with autoclaved corn cob bedding and enrichment materials. The environment was controlled at 22 ± 2 °C, 50 ± 10 % humidity, and a 12-h light/dark cycle.

JR5558 and C57BL/6J mice were anesthetized using an intraperitoneal injection of ketamine (48 mg/kg) combined with medetomidine (0.6 mg/kg). This ensured the mice were adequately sedated for the imaging procedures. One to two drops of a solution containing 1 % tropicamide and 0.5 % phenylephrine were administered to each eye to achieve proper pupil dilation, which is critical for high-quality retinal imaging.

Colour fundus imaging was performed using the Phoenix-Micron imaging system (Phoenix MICRON IV) to visualize and document the retinal lesions in the mice. Optical coherence tomography (OCT) was conducted using the Phoenix MICRON OCT2 system, providing detailed transverse images of the retinal layers and structures, which is essential for understanding the extent and nature of retinal lesions.

Fundus fluorescein angiography (FFA) was performed to assess retinal vasculature and perfusion. A 10 % fluorescein solution was prepared and administered via intraperitoneal injection. Following the injection, FFA images were captured from both eyes using the Phoenix-Micron imaging system (Phoenix MICRON IV), starting between 30 s and 2 min post-injection. This allowed for a dynamic assessment of the retinal vasculature, revealing details about blood flow and identifying any abnormalities in retinal perfusion.

The collected retinal tissues at 4 and 8 weeks of age were processed for protein extraction. Western blot analysis was performed to validate the expression levels of AKAP12, MT1G and MAFF proteins.

### 4.16 Statistical analysis

Each in *vitro* experiment was independently conducted three times from three donors, with more than six replicates per group. The western plot for mouse samples was performed using three independent samples in each group. The data are presented as means ± standard error of mean (SEM). Statistical analysis was performed using Welch's *t*-test (two-sided) between the control group (used consistently across all pairwise comparisons) and the respective treatment groups (GraphPad Prism version 10.0.2 for Windows, GraphPad Software, Boston, Massachusetts USA). Assumptions of normality were assessed using the Shapiro-Wilk test. A significance level of p < 0.05 was adopted to indicate statistical significance.

## CRediT authorship contribution statement

**Ting Zhang:** Writing – original draft, Visualization, Supervision, Methodology, Investigation, Funding acquisition. **Kaiyu Jin:** Writing – original draft, Visualization, Investigation. **Shaoxue Zeng:** Investigation. **Penghui Yang:** Visualization, Investigation. **Meidong Zhu:** Writing – review & editing, Investigation. **Jialing Zhang:** Investigation. **Yingying Chen:** Investigation. **Sora Lee:** Investigation. **Michelle Yam:** Investigation. **Yue Zeng:** Investigation. **Xiaoyan Lu:** Investigation. **Lipin Loo:** Writing – review & editing, Investigation. **G. Gregory Neely:** Supervision. **Andrew Chang:** Supervision. **Fanfan Zhou:** Writing – review & editing, Investigation. **Jianhai Du:** Writing – review & editing, Supervision, Investigation, Funding acquisition. **Xiaohui Fan:** Writing – review & editing, Supervision, Methodology, Funding acquisition, Conceptualization. **Ling Zhu:** Supervision, Project administration, Methodology, Funding acquisition, Conceptualization. **Mark C. Gillies:** Writing – review & editing, Supervision, Funding acquisition, Conceptualization.

## Declaration of competing interest

I, Ling Zhu, on behalf of all co-authors, hereby declare that they have no conflicts of interest related to our manuscript titled “Divergent Redox Responses of Macular and Peripheral Müller Glia: Implications for Retinal Vulnerability”. No financial relationships have influenced the research, analysis or conclusions presented in this manuscript.

## Figures and Tables

**figs1:**
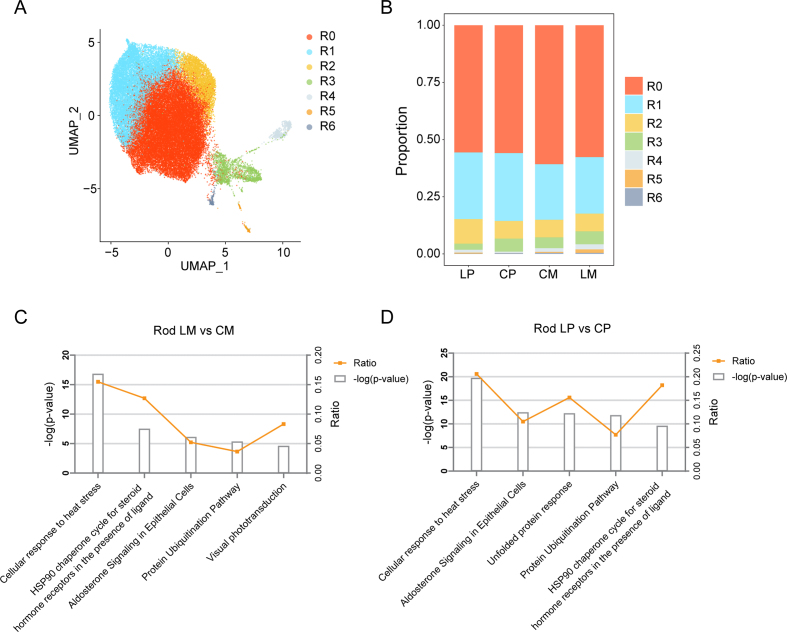


## Data Availability

Data will be made available on request.
