# Supplementary material for: Divergent redox responses of macular and peripheral Müller Glia: Implications for retinal vulnerability
Source: Redox Biol. 2025 May 24;85:103691. doi: 10.1016/j.redox.2025.103691 (PMC12178937; doi:10.1016/j.redox.2025.103691)
Supplement: Multimedia component 7 [file mmc7.docx]

**Supplementary Figures**

**
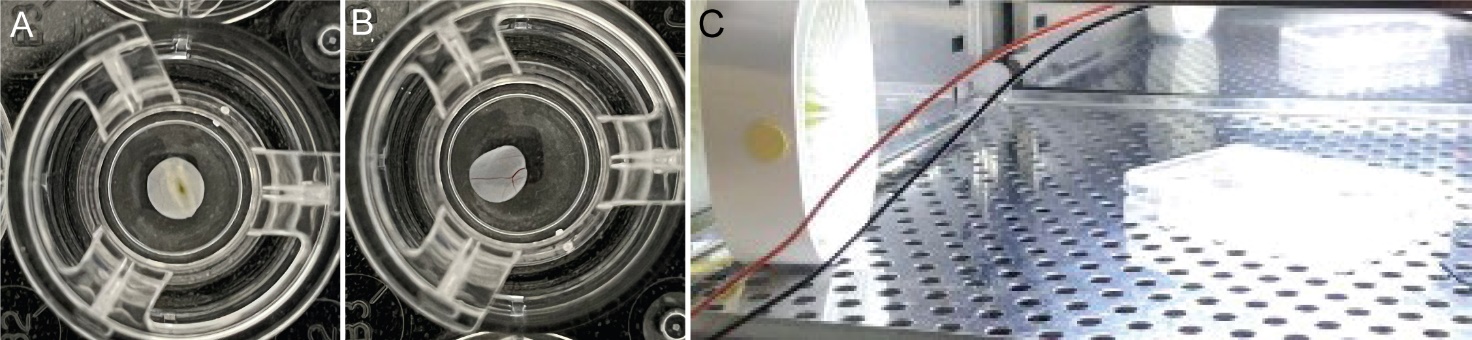
**

**Supplementary Figure 1. The light stress model of human postmortem macular and peripheral neural explants.** The human macular retinal explant (**A**) and mid-peripheral retinal explant (**B**) cultured on transwells. **C**. Light exposure on the retinal explants in transwell plates in a cell culture incubator with temperature control and ventilation.


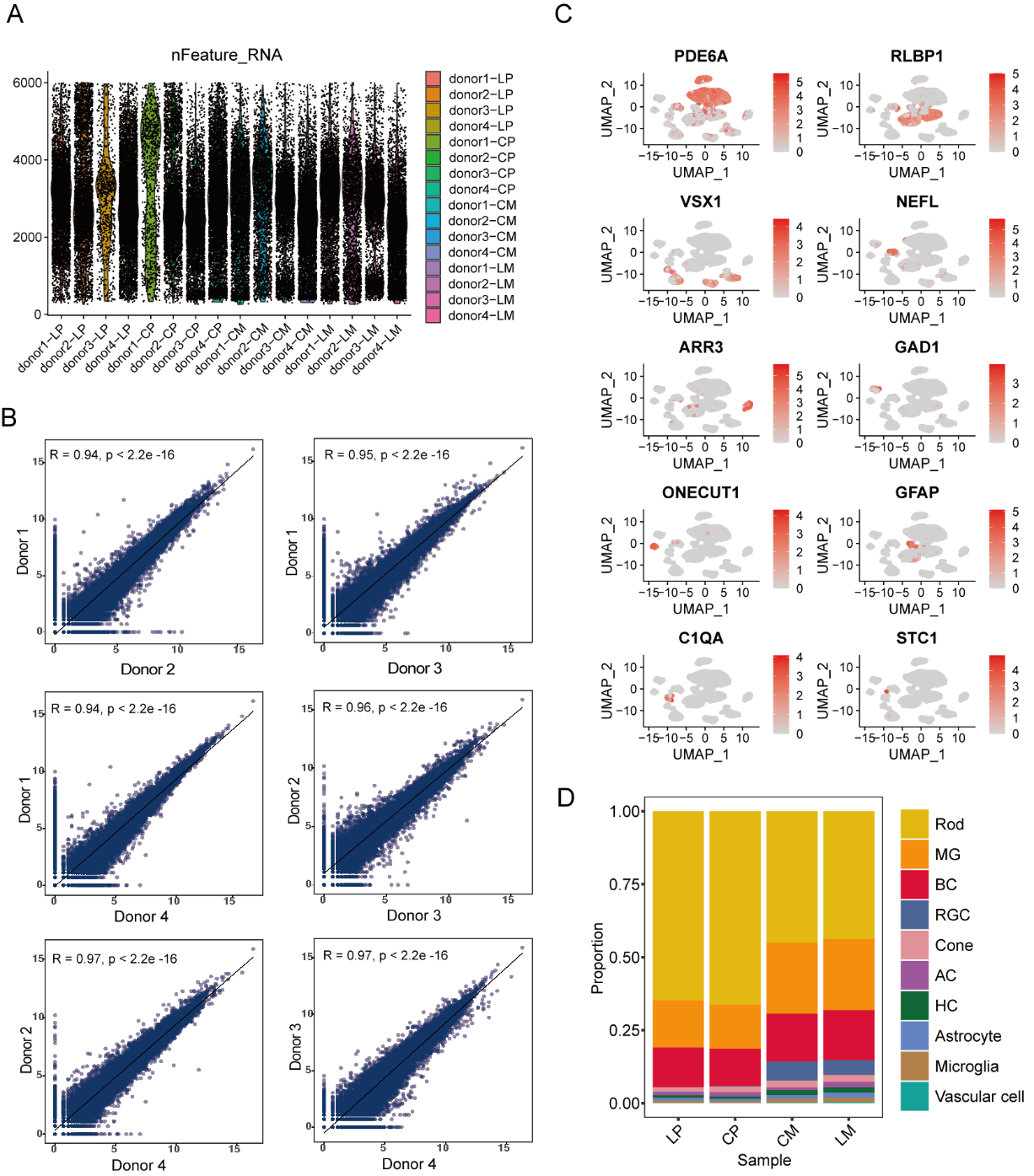


**Supplementary Figure 2. Sequencing depth and biological differences across four donor retinas**. **A**. Average number of detected RNA features per cell for each sample. **B**. Correlations of overall gene expression patterns among all donor retinas. **C**. UMAP analysis showing retinal cell markers across different cell types. **D**. Proportions of retinal cell types in different treatment groups.


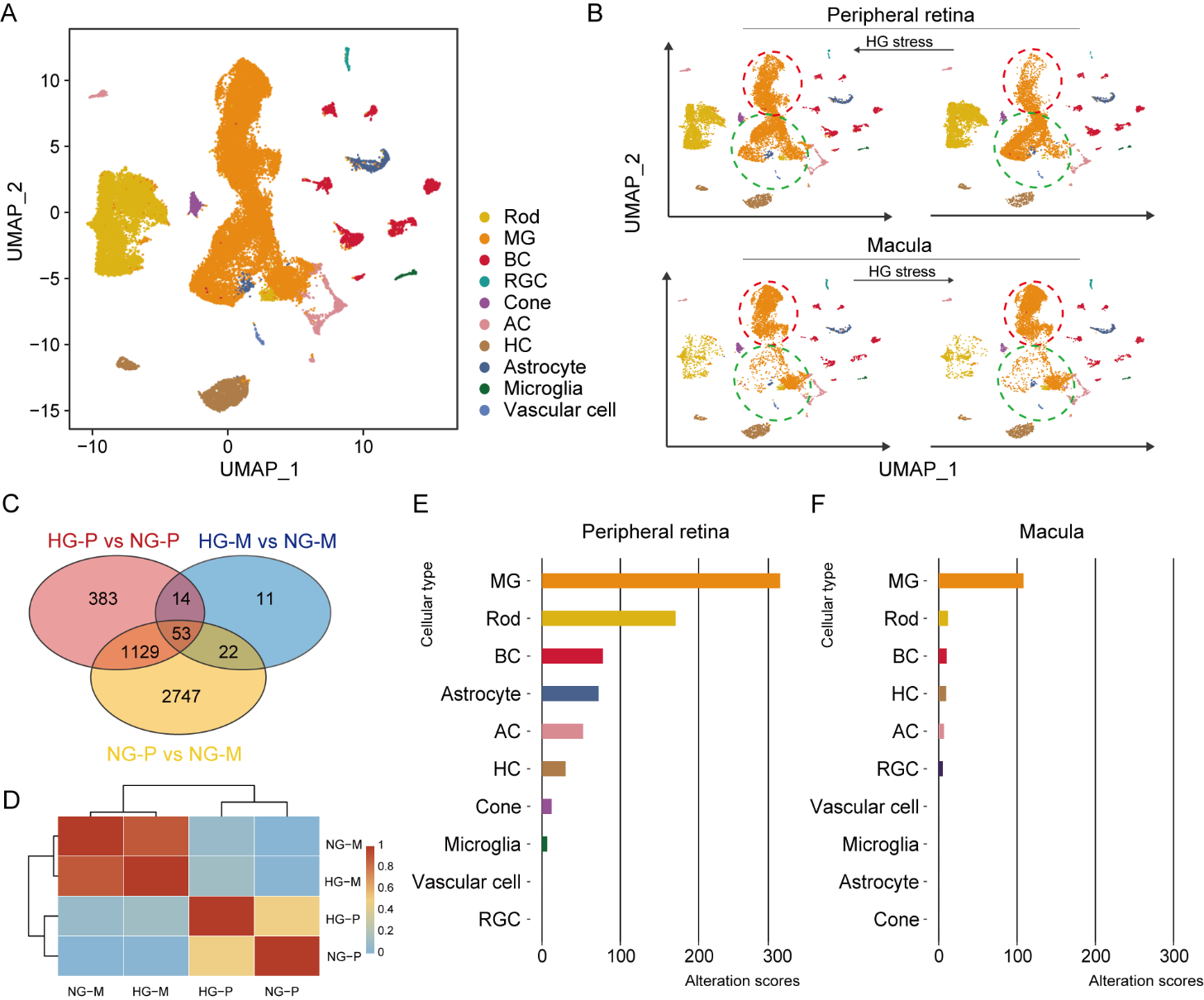


**Supplementary Figure 3. Differences between the macula and peripheral retinas with or without hyperglycaemic stress.** To determine if the findings from light-stressed retinas reflect a broader stress response, we conducted a pilot single-cell RNA sequencing (scRNA-seq) study on human maculas (**M**) and peripheral retinas (**P**) cultured *ex vivo* under hyperglycemic (**HG**) stress or normal glucose (**NG**) as a control. We identified 38,761 single cells and 10 retinal cell types from macular and peripheral retinal explants cultured in normal or high glucose conditions. The 24-h incubation may differentially affect cell survival and dissociation—rods appeared more vulnerable—resulting in fewer rod libraries and a relative enrichment of Müller-glia libraries. **A**. Uniform manifold approximation and projection (UMAP) visualization of 38,761 single cells from macular and peripheral retinal explants cultured in normal and high glucose conditions. **B**. Two major subtypes of Müller glia (MG) (orange) with distinct transcriptomic profiles, one dominant in the human macula (red circle) and the other in the peripheral retina (green circle). **C**. Venn diagram showing the number of significantly differentially expressed genes (DEGs) among treatment groups. **HG-P**: peripheral retina with high glucose; **NG-P**: peripheral retina with normal glucose; **HG-M**: macula with high glucose; **NG-M**: macula with normal glucose. **D**. Heatmap illustrating correlations between the different treatment groups. High correlation (red) in NG-M vs. HG-M represents fewer transcriptional changes; low correlation (yellow) in NG-P vs. HG-P represents significant transcriptomic profile changes. **E** & **F**. Alteration scores of different retinal cell types in the peripheral retina (**E**) and macula (**F**) in response to high glucose. A higher alteration score indicates more significant transcriptional changes.


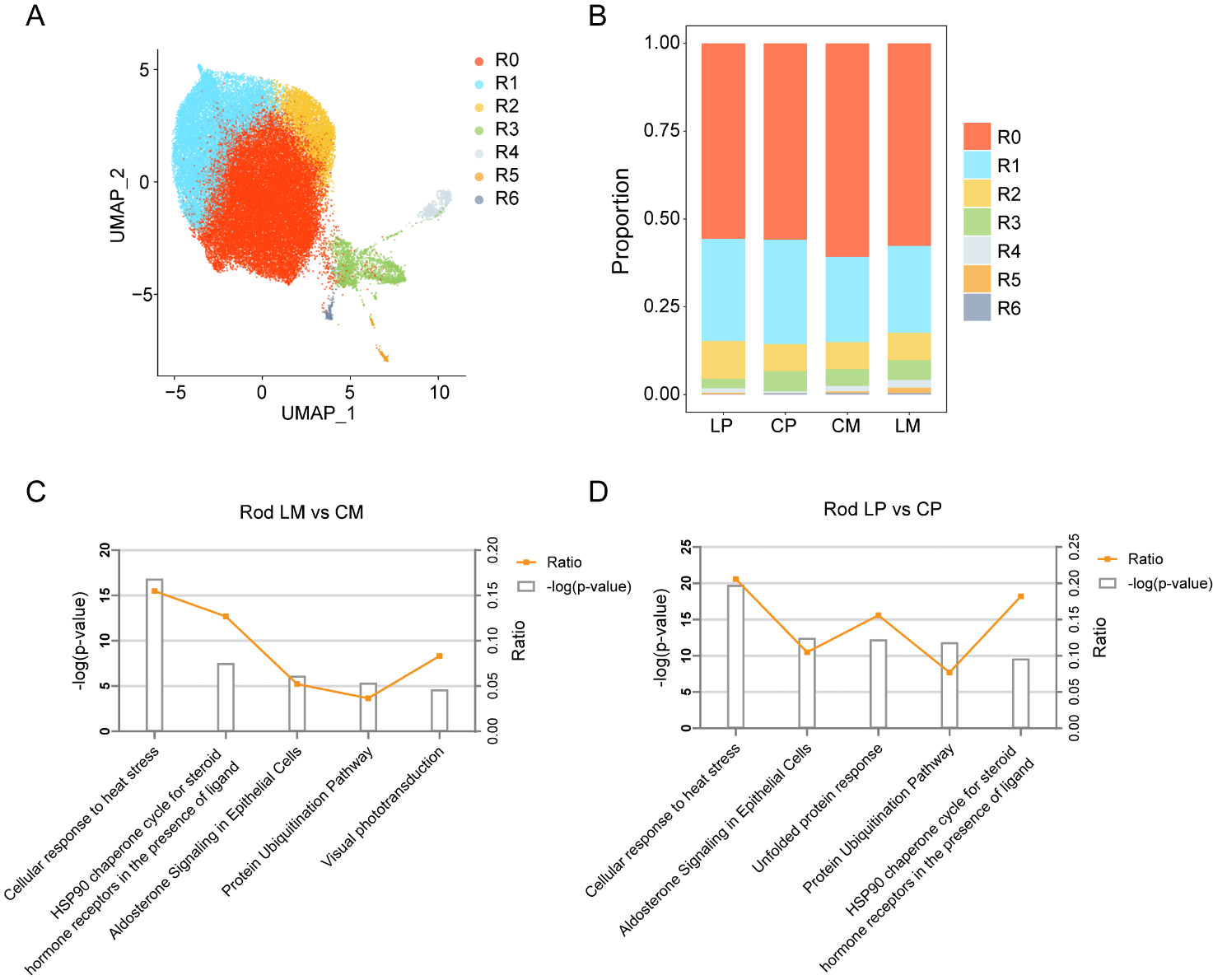


**Supplementary Figure 4. Analysis of rod subtypes and their proportions, along with** **Ingenuity Pathway Analysis (IPA) comparisons between LM vs. CM and LP vs. CP in rods. A.** UMAP visualization of rod subtypes. **B**. Proportions of different rod subtypes. **C**. IPA comparing LM and CM within rod subtypes. **D**. IPA comparing LP and CP within rod subtypes.

**
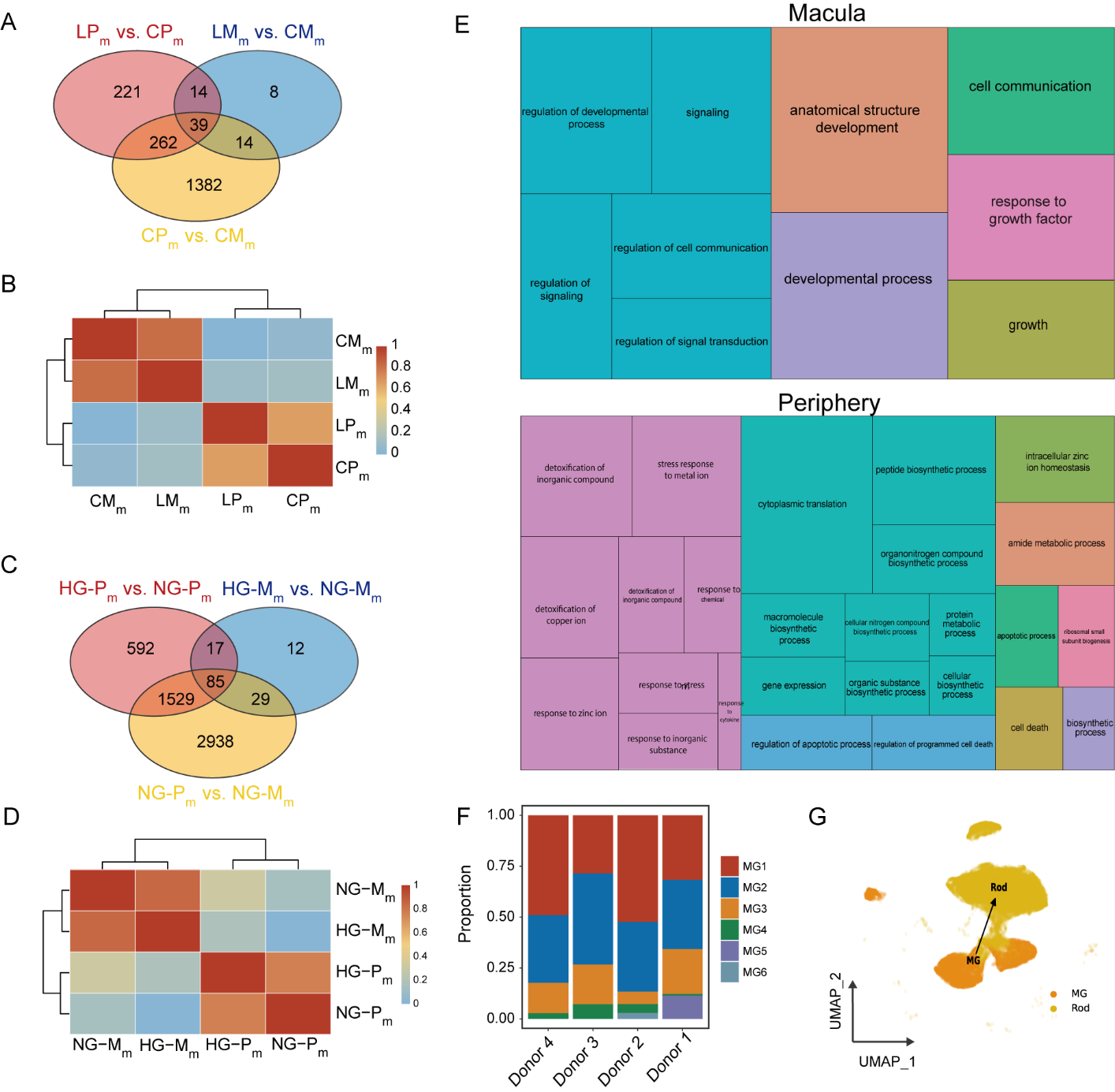
Supplementary Figure 5.** **Comparison of transcriptomic changes in Müller glia: Peripheral vs. Macular in response to light and hyperglycaemic stress**. We investigated the DEGs between total Müller glia from the macula and peripheral retina after exposure to light stress or hyperglycaemic stress across the four treatment groups. **A**. Venn diagram showing significant DEGs among treatment groups with or without light stress. **B**. Correlation analysis on transcriptomes in all Müller glia of four treatment groups in response to light stress. **LP_m_**: Light-stressed Peripheral Müller glia, **CP_m_**: Control Peripheral Müller glia, **LM_m_**: Light-stressed Macular Müller glia, **CM_m_**: Control Macular Müller glia. **C**. Venn diagram showing significant DEGs among the treatment groups with or without hyperglycaemic stress. **D**. Correlation analysis on transcriptomes in all Müller glia of four treatment groups in response to high glucose. **HG-P_m_**: peripheral Müller glia with high glucose; **NG-P_m_:** peripheral Müller glia with normal glucose; **HG-M_m_**: macular Müller glia with high glucose; **NG-M_m_**: macular Müller glia with normal glucose. **E**. Treemap of GO analysis on biological processes in human macular and peripheral Müller glia. **F**. Proportions of subtypes of Müller glia in different donors. **G**. Partition-based graph abstraction depicting the direction of differentiation from MG to rods.

**
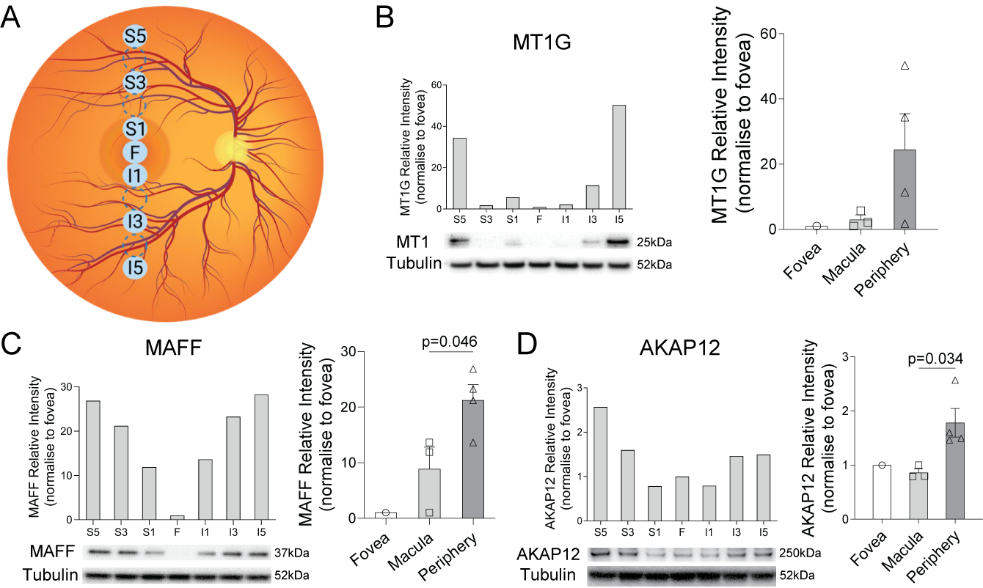
**

**Supplementary Figure 6**. **Topographic protein expressions of MT1, MAFF and AKAP12 in human postmortem retina. A**. Schematic of retinal topographic punches in the macula and peripheral retina. Solid circles ● represent 2mm-diameter retinal punches used for protein validation, while dotted circles ◌ represent 2mm-diameter retinal punches not used for protein validation. **B-D**. Protein expressions of MT1G, MAFF and AKAP12 in different locations of the neural retina. S: superior, I: inferior, F: fovea.

**
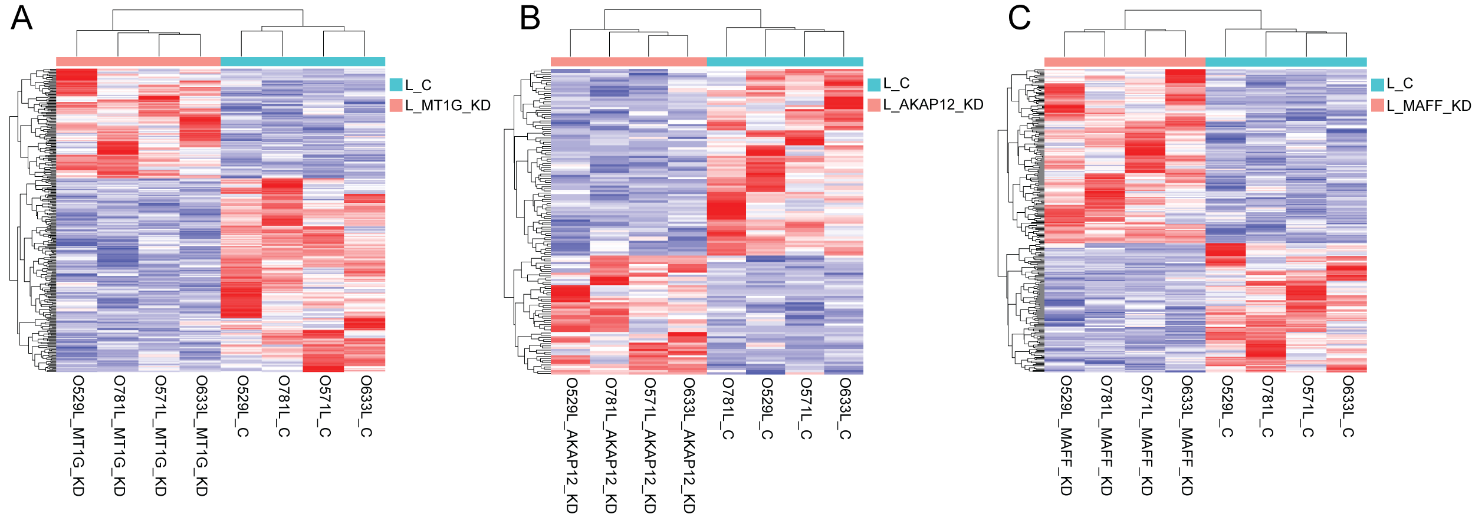
**

**Supplementary Figure 7. Heatmaps of gene expressions in in human primary Müller glia with AKAP12, MAFF and MT1G siRNA knockdown or the control group under light stress. A-C.** Heatmaps of gene expression and clustering of individual samples between AKAP12, MAFF and MT1G siRNA knockdown and control groups.

**
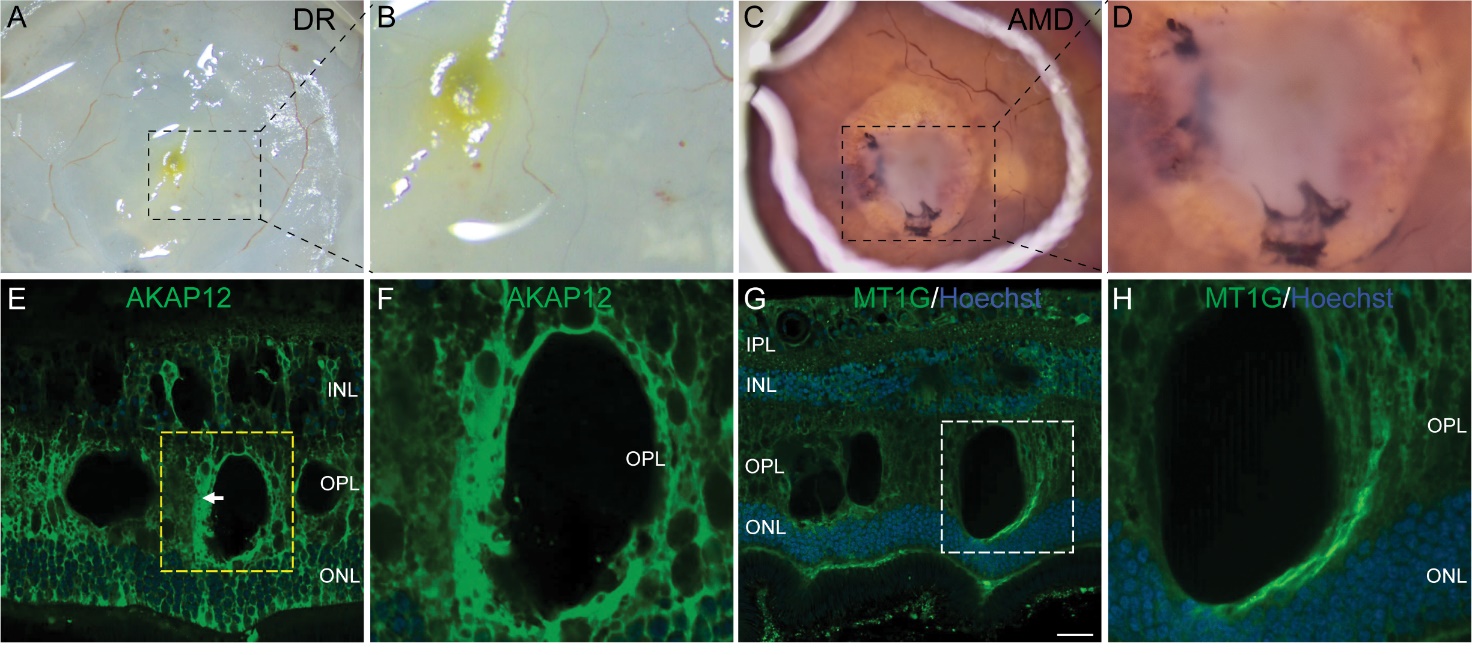
**

**Supplementary Figure 8**. **Postmortem retinas with Diabetic Retinopathy (DR) and dry age-related macular degeneration (AMD) and Activation of AKAP12 and MT1 in the retina with DR.** **A**. The fundus photo of the retina with DR. **B**. Magnified image in the dotted box of **A**. **C**. The fundus photo of the retina with dry AMD. **D**. Magnified image in the dotted box of **C**. **E**. Immunofluorescent (IF) staining of AKAP12 (green) and Hoechst (blue) on the doner retina with DR. **F**. Magnified image in the yellow dotted box of **E**. **G**. IF staining of MT1 (green) and Hoechst (blue) on the donor retina with DR. **H**. Magnified image in the white dotted boxes of **G.**
