# Supplementary material for: Divergent redox responses of macular and peripheral Müller Glia: Implications for retinal vulnerability
Source: Redox Biol. 2025 May 24;85:103691. doi: 10.1016/j.redox.2025.103691 (PMC12178937; doi:10.1016/j.redox.2025.103691)
Supplement: Multimedia component 8 [file mmc8.docx]

Supplementary Table 1. Donor Information

| **Donor Number** | **Sex** | **Age** | **Postmortem** |
| --- | --- | --- | --- |
| Donor 1 | F | 57y | 18h |
| Donor 2 | F | 63y | 20h |
| Donor 3 | F | 70y | 5h |
| Donor 4 | F | 75y | 20h |
| **Selection Criteria**   - post-mortem delay <20h - less than 75 years old - no eye conditions, diabetes, jaundice - no multi-organ failure, head trauma | | | |

# **Supplementary Table 2. scRNAseq Data - Average Expression of Target Genes**

| Gene | LP | CP | CM | LM |
| --- | --- | --- | --- | --- |
| AKAP12 | 33.9 | 28.2 | 13.9 | 14.2 |
| MAFF | 10.0 | 9.0 | 2.8 | 2.7 |
| MT1G | 40.0 | 29.1 | 5.9 | 11.7 |
| MT1E | 28.1 | 21.5 | 7.3 | 13.4 |
| GFAP | 0.9 | 1.0 | 0.8 | 0.7 |
| RLBP1 | 10.9 | 12.7 | 13.4 | 10.9 |

# **Supplementary Table 3. Ratio of Target Genes Normalized to GFAP**

| Gene | LP | CP | CM | LM |
| --- | --- | --- | --- | --- |
| AKAP12 | 39.8 | 33.1 | 16.3 | 16.6 |
| MAFF | 10.4 | 9.3 | 2.9 | 2.9 |
| MT1G | 52.4 | 38.1 | 7.8 | 15.3 |
| MT1E | 40.2 | 30.8 | 10.5 | 19.1 |

Supplementary Table 4. Donor Information

| **Donor Number** | **Sex** | **Age** | **Postmortem** |
| --- | --- | --- | --- |
| Donor 5 | F | 76y | 22h |
| Donor 6 | F | 53y | 18h |
| Donor 7 | F | 38y | 21h |
| **Selection Criteria** | | | |
| •         post-mortem delay <24h | | | |
| •         less than 80 years old | | | |
| •         no eye conditions, diabetes, jaundice | | | |
| •         no multi-organ failure, head trauma | | | |
